# Dysfunctional Crohn’s Disease-Associated NOD2 Polymorphisms Cannot be Reliably Predicted on the Basis of RIPK2 Binding or Membrane Association

**DOI:** 10.3389/fimmu.2015.00521

**Published:** 2015-10-08

**Authors:** Rhiannon Parkhouse, Tom P. Monie

**Affiliations:** ^1^Department of Biochemistry, University of Cambridge, Cambridge, UK; ^2^Department of Veterinary Medicine, University of Cambridge, Cambridge, UK; ^3^Medical Research Council Human Nutrition Research, Cambridge, UK

**Keywords:** Crohn’s disease, inflammation, innate immunity, NLR, NFκB, signal transduction, membrane localization, RIP2

## Abstract

Polymorphisms in NOD2 represent the single greatest genetic risk factor for the development of Crohn’s disease. Three different non-synonomous NOD2 polymorphisms – R702W, G908R, and L1007fsincC – account for roughly 80% of all NOD2-associated cases of Crohn’s disease and are reported to result in a loss of receptor function in response to muramyl dipeptide (MDP) stimulation. Loss of NOD2 signaling can result from a failure to detect ligand; alterations in cellular localization; and changes in protein interactions, such as an inability to interact with the downstream adaptor protein RIPK2. Using an overexpression system, we analyzed ~50 NOD2 polymorphisms reportedly connected to Crohn’s disease to determine if they also displayed loss of function and if this could be related to alterations in protein localization and/or association with RIPK2. Just under half the polymorphisms displayed a significant reduction in signaling capacity following ligand stimulation, with nine of them showing near complete ablation. Only two polymorphisms, R38M and R138Q, lost the ability to interact with RIPK2. However, both these polymorphisms still associated with cellular membranes. In contrast, L248R, W355stop, L550V, N825K, L1007fsinC, L1007P, and R1019stop still bound RIPK2, but showed impaired membrane association and were unable to signal in response to MDP. This highlights the complex contributions of NOD2 polymorphisms to Crohn’s disease and reiterates the importance of both RIPK2 binding and membrane association in NOD2 signaling. Simply ascertaining whether or not NOD2 polymorphisms bind RIPK2 or associate with cellular membranes is not sufficient for determining their signaling competency.

## Introduction

Nucleotide oligomerization domain 2 (NOD2) is a cytoplasmic pattern recognition receptor that has been connected with a variety of inflammatory disorders including Crohn’s disease, Blau syndrome, asthma, sarcoidosis, and arthritis (1). NOD2 is organized into three primary functional domains: an N-terminal effector region consisting of two caspase activation domains (CARDs); a central nucleotide-binding domain; and a C-terminal series of leucine-rich repeats (LRRs). NOD2 is maintained in an inactive state in the cell by interactions with chaperones, such as HSP90 and SGT1 (2, 3). However, detection of muramyl dipeptide (MDP), a component of bacterial peptidoglycan, along with binding and hydrolysis of ATP initiates receptor activation. Formation of the active signaling complex, most likely occurs at cellular membranes and signal propagation requires interaction with specific adaptor proteins. This primarily involves CARD-mediated interaction with the adaptor kinase receptor-interacting protein kinase 2 (RIPK2), but other proteins, such as CARD9 and TRAF4, can also be involved. This stimulates a pro-inflammatory immune response via nuclear factor kappa B (NFκB) and mitogen-associated protein kinase (MAPK) pathways.

Crohn’s disease is a chronic inflammatory bowel disorder in which intestinal immune responses, most commonly in the ileum and colon, become dysregulated. The etiology of Crohn’s disease is complex and whilst the precise cause, or causes, is uncertain, it is clear that genetic factors are an important contributory component to disease progression. Of the more than 70 genes associated with Crohn’s disease, polymorphisms in *NOD2* remain the single greatest genetic risk factor (4, 5). Three non-synonomous polymorphisms – R702W, G908R, and L1007fsincC – account for around 80% of all *NOD2*-polymorphism associated cases of Crohn’s disease reported, with homozygotic mutation resulting in around a 10-fold greater risk than heterozygotic mutation. Somewhat counter-intuitively all three of the major *NOD2* polymorphisms associated with Crohn’s disease result in a loss of receptor function and reduced inflammatory signaling (6, 7). The reduction in NOD2 function has, however, been proposed to contribute to disease via a number of different mechanisms that includes: disruption of the host microbiota, dysregulation of intestinal tolerance, and enhanced activation of other pro-inflammatory signaling pathways (8–11). The precise contribution of these mechanisms to disease pathogenesis, however, remains enigmatic.

Genetic studies have reported numerous other polymorphisms in *NOD2* that associate with Crohn’s disease. We were interested in seeing whether these polymorphisms also displayed a loss of function in response to ligand stimulation, and in identifying whether or not dysfunction could be related to disruption of RIPK2 binding and/or membrane association. We generated over 50 NOD2 constructs containing these polymorphisms and assessed their response to ligand stimulation. Twenty-three variants showed a significant reduction in signaling capacity with nine of them (R38M, R138Q, L248R, W355stop, L550V, E825K, L1007fsinC, L1007P, and R1019stop) showing a near complete loss of signaling capacity. Whilst no single cause for the loss of function could be identified, both RIPK2 binding and membrane association were important factors. Overall our data is consistent with the view that Crohn’s disease-associated NOD2 polymorphisms result in receptor dysfunction, but that the causes of this dysfunction are multi-factorial.

## Materials and Methods

### Chemicals, Plasmids, Antibodies, and General Methods

Chemical reagents were obtained from Sigma-Aldrich, UK, unless otherwise specified. HEK293T and HeLa cells were maintained in DMEM supplemented with 10% fetal calf serum, 100 μg/ml penicillin/streptomycin and 2 mM l-glutamine at 37°C and 5% CO_2_. All transfections were performed using jetPEI™(Polyplus-Transfection) as per the manufacturers’ instructions. pCMV-FLAG-NOD2, encoding N-terminally FLAG-tagged full length NOD2; pCI-myc-RIPK2, encoding N-terminally myc-tagged full length RIPK2; and pEF6-V5-mCARD9, encoding N-terminally V5-tagged full length murine CARD9 were kind gifts from Professors Thomas Kufer (12), Kate Fitzgerald, and David Underhill, respectively. Crohn’s disease-associated single nucleotide polymorphisms (SNPs) were identified using published literature (5, 6, 13–18) and the NCBI SNP database and generated using site directed mutagenesis. Mutant sequences were verified by DNA sequencing of the entire open reading frame. Plasmids encoding Firefly luciferase under the control of an NFκB (pluc) or IL-8 promoter (pluc-IL8) and Renilla luciferase controlled by a constitutive promoter (phrG) were kind gifts from Prof Clare Bryant. Antibodies used in this work were rabbit anti-FLAG (F7425, Sigma-Aldrich), mouse anti-FLAG M2 (F3165, Sigma-Aldrich), mouse anti-V5 (ab27671; Abcam), mouse anti-GAPDH (ab9485, Abcam), rabbit anti-Myc (ab9106, Abcam), goat anti-rabbit (ab6721, Abcam), goat anti-mouse (A4416, Sigma-Aldrich), Alexa-488 goat anti-mouse (A11001, Life Technologies), and Alexa-555 goat anti-rabbit (A21428, Life Technologies).

### HEK293 Reporter Assays

HEK293T cells in 96-well plates were transfected with 2 ng pLuc or pLuc-IL-8, 1 ng phrG, 0.1 ng wild-type or mutant pCMV-FLAG-NOD2, and made up to 0.1 μg total DNA with empty plasmid. One hundred nanograms per milliliter MDP (Invivogen) was added concomitant with transfection. Cells were lysed 24 h post-transfection with 1× passive lysis buffer (Promega) and luminescence measured with a LUMIstar Luminometer (BMG Labtech). Each SNP was tested in triplicate in a minimum of three separate experiments. Reporter assays routinely show variations between experiments as a result of differences in transfection efficiency and cell passage number. In order to allow comparison between experiments, data was normalized to the signal obtained using wild-type NOD2 and expressed as a percentage of wild-type signaling activity.

Data was plotted and analyzed with GraphPad Prism 5. To correct for unequal variance, data was log transformed, then subjected to statistical analysis using a one-way analysis of variance with a Bonferroni multiple comparison *post hoc* test. Data are expressed as mean + SEM and a *p*-value of <0.05 was taken as significant.

### Immunoprecipitation

HEK293T cells seeded in 6-well plates (Costar) were transfected with 1 μg of the appropriate pCMV-FLAG-NOD2 construct and 0.5 μg pCI-myc-RIP2 and incubated overnight. Cells were lysed and incubated with Dynabeads (Life Technologies) labeled with mouse anti-FLAG (Sigma) as per the manufacturers’ instructions. Samples were denatured by the addition of SDS-loading buffer (Invivogen) and heating for 5 min at 90°C prior to separation by SDS-PAGE using 4–20% Tris-glycine gels (NuSep) for 1 h at 200 V. Proteins were transferred to polyvinylidene fluoride membranes for detection by Western Blot using the appropriate antibodies.

### Immunofluorescence

HeLa cells were seeded into 12-well plates containing a sterilized 19-mm diameter glass coverslip. Cells were transfected either with 1 μg of wild-type or mutant pCMV-FLAG-NOD2, or with 0.5 μg of each of the appropriate constructs for co-immunofluorescence studies. Cells were incubated overnight before washing (1× PBS) and fixing for 15 min (4% paraformaldehyde in PBS). Cells were washed again (1× PBS), permeabilized [0.4% Triton X-100 (VWR) in 1× PBS] for 10 min, blocked for 20 min (2.5% goat serum, 1% bovine serum albumin in 1× PBS), then incubated with appropriate primary and secondary antibodies. Cells were subsequently washed three times with 1× PBS, the second wash containing a 1:5000 dilution of 10 mg/ml Hoechst 33258 in PBS to stain the nucleus. Coverslips were mounted onto microscope slides (VWR) using Mowiol mounting solution containing 2.5% 1,4-diazabicyclo[2.2.2]octane to reduce fading. Cells were visualized using an AXIO Imager.M2 microscope (Carl Zeiss Ltd., Cambridge, UK) and images created using Image J.

### Subcellular Fractionation

This was performed using a Subcellular Protein Fractionation Kit (Perbio Science UK) as per the manufacturers’ instructions from HEK293T cells overexpressing the appropriate NOD2 mutant constructs and grown in 12-well plates. Cytosolic and membrane fractions were isolated and analyzed by SDS-PAGE and western blotting. The proportion of NOD2 associated with the membrane was calculated by densitometry using Image J (19).

### Bioinformatics

Full length human NOD2 (NP_071445.1) was used to search the non-redundant protein database at NCBI. Recovered sequences with at least 90% coverage of human NOD2 were retained and manually curated to remove proteins that were described as predicted or hypothetical, or which were clear species duplications. In the latter circumstance, the protein with the highest scoring alignment for that species was retained. In total, NOD2 sequences from 30 different species were subsequently submitted for multiple sequence alignment using MUSCLE (20). Accession codes are detailed in Figure S2 in Supplementary Material. Homology models of the NOD2 CARDS were generated using the NOD1 CARD [PDB 2DBD] as a template.

## Results

### Selection of NOD2 Single Nucleotide Polymorphisms for Study

At the outset of this study, 98 SNPs in NOD2 associated with either Blau Syndrome or Crohn’s disease were identified using published data (5, 6, 13–18) and the NCBI database of NOD2 SNPs (http://www.ncbi.nlm.nih.gov/SNP/snp_ref.cgi?locusId=64127). The Blau Syndrome-associated polymorphisms were studied, and have been reported, independently of those associated with Crohn’s disease (21). Seventeen SNPs previously shown to have an NFκB activity comparable to wild-type NOD2 (6) were excluded from further study, as were three SNPs for which alternative polymorphisms existed at the residue of interest. Nine polymorphisms could not be successfully generated in the NOD2 expression vector. In total, 53 Crohn’s disease-associated polymorphisms were taken forward for functional characterization in conjunction with wild-type NOD2 and the inactive Walker-B mutant D379A as control constructs.

### Crohn’s Disease-Associated NOD2 Polymorphisms can Result in a Defective Response to MDP Stimulation

**Figure 1:**
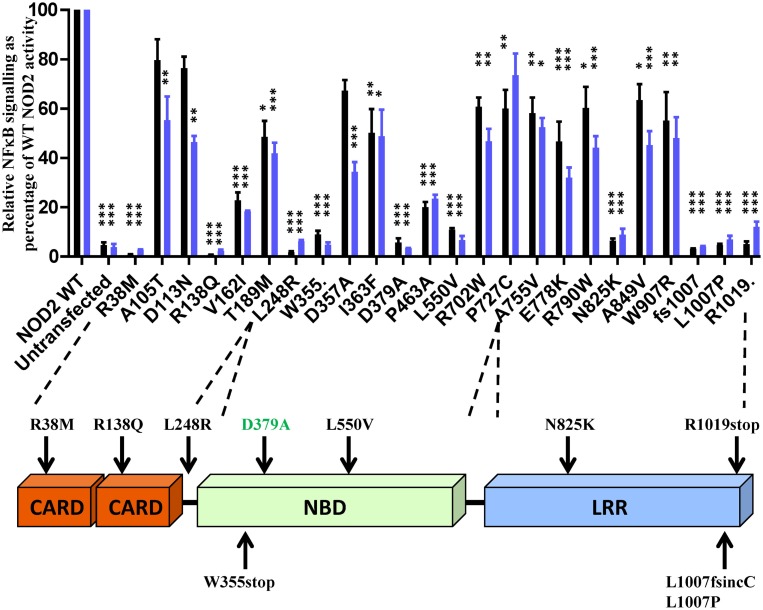
**Twenty-three NOD2 polymorphisms show a significant reduction in signaling following stimulation with MDP**. The ability of NOD2 polymorphisms to signal via NFκB (black bars) or activate the IL-8 promoter (blue bars) was determined following stimulation with 100 ng/ml MDP. Signaling functionality is expressed as a percentage of wild-type receptor activity. All polymorphisms were tested in triplicate in a minimum of three separate experiments. Error bars show SEM. Data was log transformed and statistical significance determined using one-way ANOVA with Bonferroni post-test for multiple samples. Only polymorphisms showing a significant reduction in signaling are plotted (data for the other polymorphisms is provided in Figure S1 in Supplementary Material) – *p < 0.05, **p = < 0.01, ***p = < 0.001. A schematic of the NOD2 domain structure is provided with the location of polymorphisms showing <15% activity marked.

To determine the effect of the NOD2 polymorphisms on receptor function, we assessed their ability to signal in response to MDP stimulation using NFκB and IL-8 reporter assays. Twenty-three of the polymorphisms, along with the Walker-B mutant D379A, showed a significant reduction in signaling (Figure 1). All polymorphisms that showed a significant reduction in signaling activity with the exception of A105T, D113N, D357A (IL-8 only), and P727C (NFκB only) were significantly impaired in both reporter systems. Nine of the polymorphisms (R38M, R138Q, L248R, W355stop, L550V, N825K, L1007fs, L1007P, and R1019stop) and the control D379A showed major functional impairment with signaling below 15% of wild-type NOD2 (Figure 1). None of the polymorphisms tested resulted in receptor hyperactivation in response to MDP (Figure S1 in Supplementary Material).

### Amino Acid Conservation can be Indicative but does not Correlate with Functional Impact

**Figure 2:**
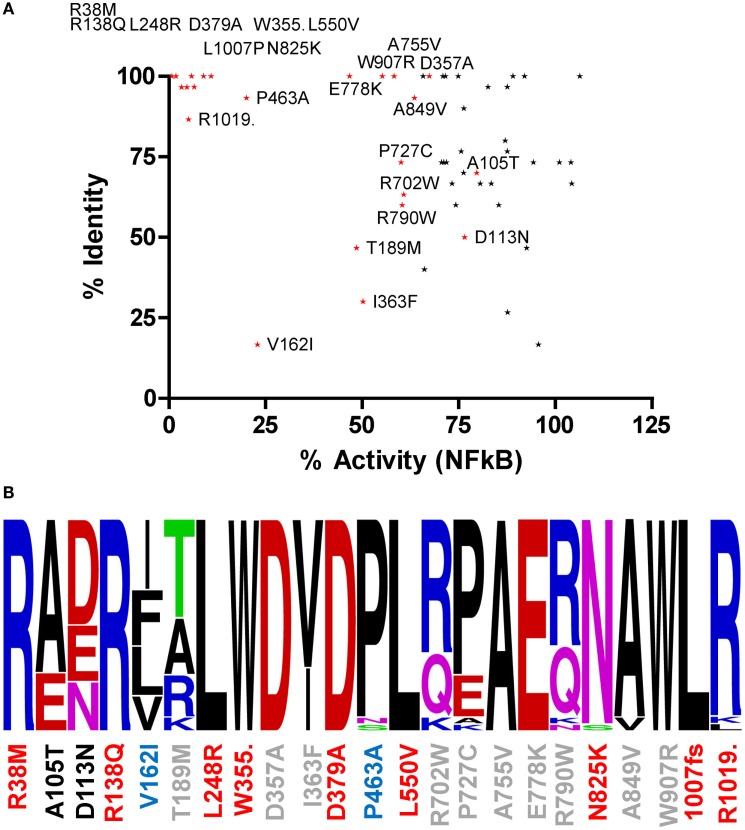
**The relationship between residue conservation and functional impact**. (**A**) Scatter plot of percentage residue identity and percentage NFκB signaling activity for all polymorphisms studied. Polymorphisms that cause a significant reduction in signaling are colored red and labeled. The points for R38M and R138Q overlap on the graph. (**B**) WebLogo representation of residue conservation for those polymorphisms producing a significant reduction in signaling. Polymorphism position is denoted on the *x*-axis and colored by level of NFκB signaling compared to WT NOD2 – red <15%, blue 15–45%, gray 46–75%, and black >75%. Amino acid residues are colored as follows: basic residues – blue; acidic residues – red; hydrophobic residues – black; polar acidic residues – green; and polar basic residues – pink.

Amino acids with important functional roles regularly show higher levels of evolutionary conservation. We updated our previous cross-species alignment of NOD2 (22) and compared the level of evolutionary conservation for each of our polymorphic NOD2 variants (Figure 2; Figures S1 and S2 in Supplementary Material). Consistent with a key functional role, nine of the polymorphisms that showed a significant reduction in signaling were completely conserved across all aligned species, and a further two (E825 and L1007) were conserved in 29/30 species (Figure 2). However, high levels of residue conservation could not be used as a direct predictor of functional impact as seven completely conserved residues showed no impact on signaling (Figure 2A). Interestingly, V162 is only conserved in five of the 30 species and despite all of the substitutions being conservative hydrophobic ones, the polymorphism V162I signals at <25% of the wild-type protein (Figures 1 and 2B). In fact 10 species, including members of the bovine, porcine, caprine, and canine families, all possess an isoleucine in this position and therefore may show impaired NOD2 functionality.

### RIPK2 Binding is not Sufficient for NOD2 Signaling

**Figure 3:**
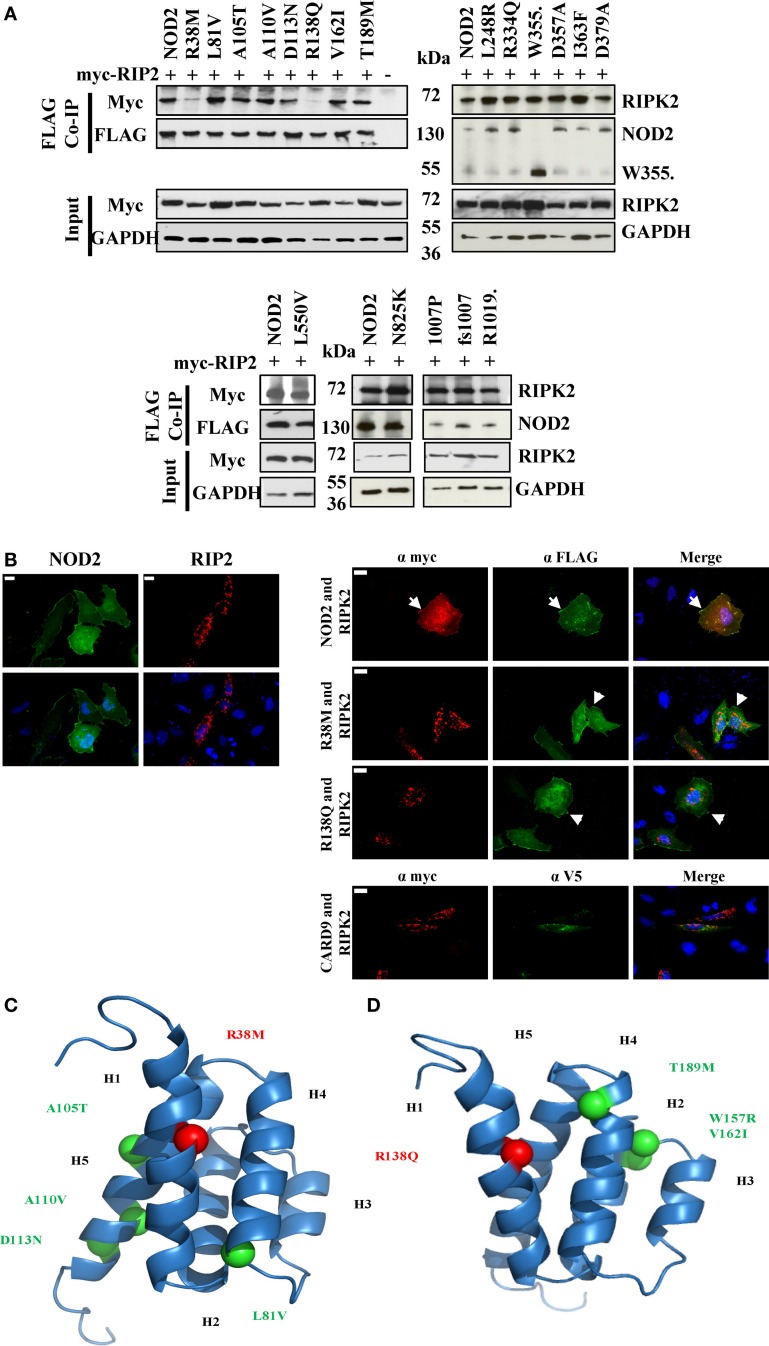
**Impact of NOD2 polymorphisms on the interaction with RIPK2**. **(A)** HEK293T cells seeded in 6-well plates were transiently transfected with 0.5 μg pCI-myc-RIP2 and 1 μg pCMV-FLAG-NOD2 polymorphic constructs as labeled. After 24 h, cell lysates were immunoprecipitated using Dynabeads labeled with mouse anti-FLAG antibody. Proteins were separated by SDS-PAGE and proteins detected using the antibodies specified. **(B)** Immunofluorescence was performed in 12-well plates using HeLa cells seeded onto coverslips using 0.5 μg pCMV-FLAG-NOD2 or pCI-myc-RIP2 and the protein localization was visualized following antibody staining (left panel). Co-localization studies were performed by transfecting 0.5 μg of pCI-myc-RIP2 with 0.5 μg of each of pCMV-FLAG-NOD2/R38M/R138Q or pEF6-V5-CARD9 (right panel). Examples of membrane-associated fluorescence are indicated by arrows. The scale bar indicates 20 μm. Cartoon representation of homology models of the first **(C)** and second **(D)** CARDs of NOD2 showing the conserved location of R38 and R138 (red spheres) and the location of other CARD located polymorphisms (light green). Helices are annotated as H1–H6.

Following activation of NOD2, signal propagation requires interaction with the adaptor protein RIPK2 in a CARD-dependent manner. To determine if NOD2 SNPs with a signaling defect were impaired in their ability to interact with RIPK2 we performed co-immunoprecipitations in HEK293T cells between FLAG-tagged NOD2 constructs and Myc-tagged RIPK2. With the exception of R38M and R138Q, all the tested NOD2 polymorphisms retained the ability to bind RIPK2 (Figure 3A; Figure S1 in Supplementary Material), thereby indicating that whilst the interaction with RIPK2 is necessary for signal transduction, it is not sufficient. This is consistent with recent observations between NOD1 and RIPK2 (23). The inability of R38M and R138Q to interact with RIPK2 was confirmed by immunofluorescence in HeLa cells (Figure 3B). Wild-type NOD2 showed membrane-based co-localization with RIPK2 following overexpression. However, whilst both R38M and R138Q still associated with the cell membrane they did not localize with RIPK2 (Figure 3B). In fact, in the presence of R38M and R138Q RIPK2 showed a punctate distribution in the cell interior (Figure 3B) that matched its distribution when transfected into HeLa cells in the absence of NOD2 (Figure 3B). Homology models of the NOD2 CARDs (Figures 3C,D) indicated that both R38 and R138 are predicted to be located in equivalent positions on the first helix of the first and second CARDs, respectively. This strongly suggests that this region on both CARDs is crucial for the interaction with RIPK2.

### Association of NOD2 Polymorphisms with Membranes does not Directly Relate to Functionality

**Figure 4:**
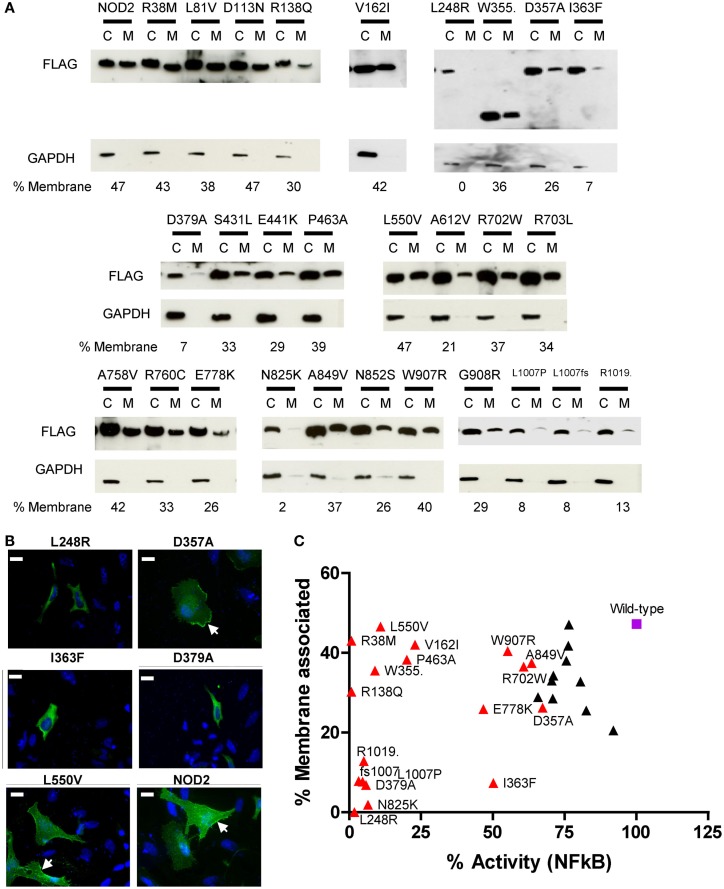
**Membrane association of NOD2 polymorphisms**. (**A**) NOD2 polymorphisms were transiently transfected in HEK293 cells. After 24 h cells were harvested and the membrane and cytoplasmic fractions separated using the Pierce Subcellular Fractionation Kit. Proteins were resolved using SDS-PAGE and NOD2 constructs detected with anti-FLAG antibody. Detection of GAPDH confirmed the relative purities of the membrane and cytoplasmic fractions. The proportion of NOD2 associated with the membrane was estimated by densitometry using ImageJ. (**B**) Representative immunofluorescence images showing the cellular localization of overexpressed NOD2 constructs in HeLa cells. One microgram of the relevant DNA was transfected into HeLa cells seeded into 12-well plates containing a coverslip. After 24 h, cells were fixed using 4% PFA in PBS and permeabilized using 0.4% Triton X-100. Proteins were stained using mouse anti-FLAG primary antibody (Sigma) and Alexa Fluor^®^ 555 goat anti-rabbit secondary antibody (Life Technologies). Cells were visulaised using an AXIO Imager.M2 microscope (Zeiss) and images created using Image J. Membrane localization is indicated by arrows and the scale bar equals 20 μm. (**C**) Scatter plot of percentage membrane association plotted against percentage NFκB signaling activity. Polymorphisms that showed a significant reduction in signaling are colored red and labeled. The wild-type receptor is represented as a purple square and labeled.

Our immunofluorescence of R38M and R138Q (Figure 3B) indicated that these polymorphisms were still recruited to the membrane despite being unable to signal. This suggested that whilst the association of NOD2 with cellular membranes is important for signal transduction, it does not in fact guarantee that signaling will occur. To study whether the other dysfunctional polymorphisms showed altered membrane association we performed immunofluorescence and subcellular fractionation on a collection of overexpressed polymorphisms displaying a range of signaling activity (Figure 4). The extent of membrane association was estimated by densitometry of the subcellular fractionation data (Figure 4A). As shown by the scatter plot in Figure 4C, no clear correlation between the degree of membrane association and the extent of receptor signaling could be drawn. However, polymorphisms associated with a significant reduction in signaling could be assigned into three main groups. Those with a membrane association and signaling activity below 15% (L248R, D379A, N825K, L1007P, L1007fs, and R1019stop); those with membrane association similar, or fractionally lower than wild-type and low signaling activity (R38M, R138Q, V162I, W355stop, P463A, and L550V); and those with slightly impaired signaling, but similar or slightly low membrane association. With the exception of I363F none of the polymorphisms with <20% membrane association signaled at levels above 15% of the wild-type receptor. Analysis of a wider range of functional and dysfunctional NOD2 polymorphisms is needed before it can be determined whether this is a strict correlation. The association of R38M, R138Q, and W355stop at levels broadly similar to wild-type NOD2 suggest that whilst membrane association is likely to play an important role in NOD2 signaling, it is not sufficient for signal transduction to occur.

## Discussion

The etiology of Crohn’s disease is complex and is influenced by combinations of genetic, lifestyle, and environmental factors. Polymorphisms in NOD2 present the strongest genetic susceptibility factor although over 70 separate genes have been reported to be associated with the risk of disease development and progression (4). Our understanding of how NOD2 SNPs predispose to Crohn’s disease is improving and in general, they appear to result in defective NOD2 function (10). However, the extent and severity of this defect is variable and appears to show an element of cell-type and/or stimuli specificity. In this work, we have analyzed 53 Crohn’s disease-associated SNPs for their effect on three key functional consequences of NOD2 stimulation, namely: basal and ligand-induced NFκB-driven signaling, interaction with RIPK2, and membrane localization. Due to the low population frequency of the SNPs studied, we employed an overexpression system, which despite their acknowledged shortcomings, still provide a valuable system for understanding the functional impact of mutations on protein function.

It is clear from our results, and in agreement with the work of other researchers investigating different NOD2 polymorphisms, that not all Crohn’s disease-associated NOD2 polymorphisms result in the same degree of functional impact. Indeed, only 23 of the SNPs we tested showed significant functional impairment. This may reflect the presence of subtle functional impacts that are masked by protein overexpression; that the polymorphisms affect functions not tested here, such as signaling via CARD9; or that their reported association with Crohn’s disease is artifactual. These are all elements that require further study.

Earlier studies have implicated R38, following mutation to alanine, as important for the interaction of NOD2 and RIPK2 (24). Our observations, in which R38M and R138Q were the only polymorphisms to lose the ability to efficiently interact with RIPK2, are consistent with this assertion and also suggest a crucial role in RIPK2 binding for R138Q. Homology models show that R38 and R138 occupy equivalent position in the first helix of the first and second CARDs, respectively (Figures 3C,D) in a region that forms part of a basic patch on the surface of each CARD. We observed dramatic reductions in signaling capacity for R38M and R138Q, with the latter observation consistent with earlier work (6). That other signaling defective NOD2 polymorphisms retain the ability to interact with RIPK2 is consistent with our recent observations between NOD1 and RIPK2 (23) and confirms that for both NOD1 and NOD2, the engagement of RIPK2 is necessary, but not sufficient for signal propagation.

It has been widely reported that membrane association is an integral requirement for NOD2 signaling (17, 25–27). Our observations in this work suggest that the connection between NOD2 signaling and membrane association requires further clarification. Five polymorphisms (L248R, N825K, L1007P, L1007fs, and R1019stop) plus the Walker-B mutant (D379A) showed minimal capacity to signal following ligand stimulation and had very low levels of membrane association. Whilst I363F despite showing <10% membrane association, still signaled at around half the level of wild-type protein. In contrast, other polymorphisms showed low (V162I and P463A) or very low (R38M, R138Q, W355stop, and L550V) levels of activity, but have levels of membrane association little different to wild-type NOD2. In the case of R38M and R138Q, the lack of signaling clearly relates to their failure to recruit RIPK2. However, V162I, W355stop, P463A, and L550V all associate with membrane and all engage RIPK2, but are still heavily impaired in their signaling capacity, suggesting that the reasons for NOD2 dysfunction in these cases are somewhat more complex and elusive.

Although residue conservation does not provide a clear and definitive correlation with the functional impact of the polymorphisms, it can help rationalize why certain residues do, or do not, have functional consequence when mutated. For example, amino acids that showed high levels of evolutionary variation, such as W157, R235, R708, and R760, generally showed limited impairment in signaling. Whereas, the functional impact of polymorphic changes at highly conserved residue locations, such as R38, R138, L248, and L1007, was much more dramatic. In some instances, the reason for the dramatic impact and hence evolutionary conservation are clear. For example, R38 and R138 are needed for interaction with RIPK2; N825 forms part of the LRR asparagine ladder and hence contributes to structural integrity; and L1007 and R1019 are in the C-terminus of the protein and important for membrane localization. On the other hand, why L248 and L550 are completely conserved and functionally crucial is less apparent. For L248, its importance may relate to its proximity to CARD9 (28) and TRAF4 (29) binding sites and the introduction of a large basic amino acid in the polymorphism may disrupt these interactions. Similarly, mutation of L550 could result in destabilization of the protein structure or interfere with the interaction with chaperone proteins and hence disrupt the transition from the inactive to active conformation of NOD2. Reasons for the lack of functional impact for polymorphisms of the other highly conserved residues are less clear, but in at least the case of N852S, this reflects a change of residue that is permitted in the structural context of that amino acid, i.e., serine can functionally substitute for asparagines in the LRR asparagine ladder (30).

Our work here provides one of the most detailed investigations into the functional impact of Crohn’s diseases-associated polymorphisms. By assessing not just receptor signaling, but also the interaction with RIPK2, evolutionary conservation, and receptor localization, we have provided an important insight into the diverse range of functional impacts displayed by these polymorphisms. Importantly, the work highlights the benefit of studying the functional impact of polymorphic variation through highlighting the complex relationship between NOD2 and its association with RIPK2 and cellular membranes in relation to signal transduction.

## Author Contributions

RP performed and analyzed the data, drafted the manuscript, and approved the final version; TM conceived the study, analyzed the data, wrote and revised the manuscript, and approved the final version.

## Conflict of Interest Statement

The authors declare that the research was conducted in the absence of any commercial or financial relationships that could be construed as a potential conflict of interest.

## Supplementary Material

The Supplementary Material for this article can be found online at http://journal.frontiersin.org/article/10.3389/fimmu.2015.00521

## Abbreviations

CARD, caspase activation domain; LRR, leucine-rich repeat; MDP, muramyl dipeptide; NFκB, nuclear factor κB; NOD2, nucleotide oligomerization domain 2; RIPK2, receptor-interacting serine/threonine kinase 2; SNP, single nucleotide polymorphism.
