# Supplementary material for: Dysfunctional Crohn’s Disease-Associated NOD2 Polymorphisms Cannot be Reliably Predicted on the Basis of RIPK2 Binding or Membrane Association
Source: Front Immunol. 2015 Oct 8;6:521. doi: 10.3389/fimmu.2015.00521 (PMC4597273; doi:10.3389/fimmu.2015.00521)
Supplement: Supplementary file 1 [file Data_Sheet_1.DOCX]

| SNP | NFκB | IL-8 | Conservation (30 species) | RIPK2 | Expression | Localisation  (% membrane) |
| --- | --- | --- | --- | --- | --- | --- |
| R38M | 0.7% | 2.8% | R=30 | NO | ++ | M (43) |
| L81V | 75.6% | 66.2% | L=23,I=6, -=1 | + | ++ | M (38) |
| A105T | WT | WT | A=21,E=8, -=1 | + |  |  |
| A110V | WT | WT | A=22,V=7, E=1 | + |  |  |
| D113N | 76.4% | 43.7% | D=15,E=8, N=7 | + | ++ | M (47) |
| R138Q | 0.7% | 2.4% | R=30 | NO | ++ | M (30) |
| W157R | WT | WT | R=9,Q=8, L=6,W=5, V=2 |  |  |  |
| V162I | 23.2% | 18.3% | I=10,F=8, L=7, V=5 | + | ++ | M (42) |
| T189M | 49.0% | 41.8% | T=14,A=8, R=6, K=2 | + | ++ |  |
| R235C | WT | WT | R=22,H=3, C=2, L=2 |  |  |  |
| L248R | 1.7% | 6.2% | L=30 | + | ++ | C (0) |
| E249G | WT | WT | E=23, D=7 |  |  |  |
| N289S | WT | WT | N=29, S=1 |  |  |  |
| D291N | WT | WT | D=20, E=10 |  |  |  |
| A301V | WT | WT | A=30 |  |  |  |
| Q335H | WT | 59.0% | Q=22,K=4, R=4 |  |  |  |
| W335. | 8.9% | 4.7% | W=30 | + | ++ | M (36) |
| D357A | 60% | 34.3% | D=30 | + | ++ | M (26) |
| I363F | 50% | 48.7% | V=21, I=9 | + | + | C (7) |
| D379A | 5.7% | 3.4% | D=30 | + | ++ | C (7) |
| R391C | WT | 54.7% | R=14,E=8, Q=6, H=2 |  |  |  |
| S431L | 70.5% | WT | S=22,G=7, T=1 | + | ++ | M (33) |
| E441K | 70.9% | 65.1% | E=30 | + | ++ | M (29) |
| E462K | WT | 68.3% | E=20, D=10 |  | ++ |  |
| P463A | 18.6% | 23.4% | P=28,N=1, S=1 | + | ++ | M (39) |
| L550V | 10.9% | 6.6% | L=30 | + | ++ | M (47) |
| A612T | 74.8% | 62.6% | A=30 |  |  |  |
| A612V | WT | WT | A=30 | + |  | M (21) |
| A660G | 76.1% | WT | A=21,-=2, E=1,G=1, K=1,M=1, T=1,V=1 |  |  |  |
| P668L | 73.2% | 66.5% | P=20,T=8, L=1, Q=1 |  | ++ |  |
| R684Q | WT | WT | Q=19,R=8, K=2, W=1 |  | + |  |
| R702W | 60.7% | 46.8% | R=19,Q=0, K=2 | + | ++ | M (37) |
| R703L | 71.1% | WT | R=22,K=6, N=1, L=1 | + |  | M (34) |
| R708H | 74.2% | WT | R=18,V=4, A=2,Q=2, G=1, S=1 |  |  |  |
| P727L | 59.9% | 73.5% | P=22,E=6, A=1, K=1 |  |  |  |
| A755V | 58.0% | WT | A=30 |  |  |  |
| A758V | WT | WT | A=27, T=3 | + |  | M (42) |
| R760C | WT | 81.1% | R=20,A=5, S=4, C=1 | + |  | M (33) |
| E778K | 46.7% | 32.0% | E=30 | + |  | M (26) |
| R790W | 60.2% | 44.13% | R=18,Q=10, N=1, K=1 |  |  |  |
| R791Q | WT | WT | R=18,N=7, Q=2,K=1, W=1, X=1 | + |  |  |
| A794P | 71.9% | 68.2% | A=22, G=8 |  |  |  |
| Q809H | WT | WT | Q=30 |  |  |  |
| Q809R | WT | WT | Q=30 |  |  |  |
| N825K | 6.5% | 8.8% | N=29, S=1 | + |  | C (2) |
| A849V | 63.4% | WT | A=28, V=2 | + |  | M (38) |
| N852S | WT | WT | N=29, -=1 | + |  | M (26) |
| A885T | WT | 68.3% | A=24,S=4, E=1, P=1 |  |  |  |
| W907R | 55.1% | 59.4% | W=30 | + |  | M (40) |
| G908R | 65.7% | 58.2% | G=30 | + |  | M (29) |
| G924D | 66.1% | WT | G=12,A=7, K=4,E=3, R=1,S=1, T=1, V=1 |  |  |  |
| L1007fsinsC | 3.2% | 4.6% | L=29, -=1 | + |  | C (8) |
| L1007P | 4.5% | 6.8% | L=29, -=1 | + |  | C (8) |
| R1019. | 5.0% | 12% | R=26,-=2, K=1, L=1 | + |  | C (12) |

**Supplementary File 1: Summary of functional impact of NOD2 polymorphisms.**

Rows are colour coded based on their activity following ligand stimulation. Light grey = > 80 % activity (i.e. approx WT); green = 50-80 % activity; purple = 15-50 % activity; pink = < 15 % activity. Activity results are shown for stimulated NFκB and IL-8 conditions. Amino acid frequency is reported in the conservation column. For interactions with RIPK2: + = preserved interaction; NO = no visible interaction; blank = not tested. For expression: ++ = comparable to wildtype; + = reduced expression. For cellular localisation: C = cytoplasmic, M = plasma membrane; blank = not tested.
