# Supplementary material for: Dysfunctional Crohn’s Disease-Associated NOD2 Polymorphisms Cannot be Reliably Predicted on the Basis of RIPK2 Binding or Membrane Association
Source: Front Immunol. 2015 Oct 8;6:521. doi: 10.3389/fimmu.2015.00521 (PMC4597273; doi:10.3389/fimmu.2015.00521)
Supplement: Supplementary file 2 [file Data_Sheet_2.DOCX]

ERE77543.1|Cricetulus_griseus --------------------------------------------------MESRGGNSSI

NP_001099642.1|Rattus_norvegicus ------------------------------------------------------------

AAN52938.1|Mus_spretus ------------------------------------------------------------

AAN52482.1|Mus_musculus_domestic ------------------------------------------------------------

AAN52483.1|Mus_musculus_castaneu ------------------------------------------------------------

Q8K3Z0.1|Mus_musculus -----------------------------------------------------------M

AAN52480.1|Mus_musculus_musculus ------------------------------------------------------------

EHB15612.1|Heterocephalus_glaber MVAGIVESGQQLSRKPRSLCLHLSEMVTGMPEALSQNNQDFLSPEGLLVWFWLPAGPFGI

KFO37911.1|Fukomys_damarensis ------------------------------------------------------------

BAH24204.1|Sus_scrofa ------------------------------------------------------------

AHL44890.1|Capra_hircus ------------------------------------------------------------

AJC01046.1|Bubalus_bubalis ------------------------------------------------------------

ELR51388.1|Bos_mutus ------------------------------------------------------------

AAS09828.1|Bison_bison ------------------------------------------------------------

AAS09827.1|Bos_indicus ------------------------------------------------------------

NP_001002889.1|Bos_taurus ------------------------------------------------------------

NP_001273968.1|Canis_lupus_famil ------------------------------------------------------------

ELK15377.1|Pteropus_alecto ------------------------------------------------------------

AAS89989.1|Saguinus_oedipus ------------------------------------------------------------

Q53B88.1|Hylobates_lar ---------------------------------------MGEEGGSVSHDEEERASVLLG

NP_071445.1|Homo_sapiens ---------------------------------------MGEEGGSASHDEEERASVLLG

NP_001098710.1|Pan_troglodytes ---------------------------------------MGEEGGSVSHDEEERASVLLG

AAY97879.1|Danio_rerio ------------------------------------------------------------

ACX71753.1|Ctenopharyngodon_idel ------------------------------------------------------------

AFY26969.1|Carassius_auratus ------------------------------------------------------------

AEG89706.1|Labeo_rohita ------------------------------------------------------------

ADV31549.1|Oncorhynchus_mykiss ------------------------------------------------------------

NP_001035913.1|Takifugu_rubripes ------------------------------------------------------------

AFV53358.1|Epinephelus_coioides ------------------------------------------------------------

AJF23836.1|Larimimichthys_crocea -------------------------------------------------MTDGRKKAGKP

ERE77543.1|Cricetulus_griseus LDTGCDMCSQEDFQAQRSQLVALLVS-GSLEGFESILDWLLSWDVLSWEDYEGLSLPGQP

NP_001099642.1|Rattus_norvegicus ------MCSQEDFQAQRSQLVALLVS-GSLEGFESILDWLLSWDVLSREDYEGLSLPGQP

AAN52938.1|Mus_spretus ------MCSQEEFQAQRSQLVALLVS-GSLEGFESILDWLLSWDVLSREDYEGLSLPGQP

AAN52482.1|Mus_musculus_domestic ------MCSQEEFQAQRSQLVALLIS-GSLEGFESILDWLLSWDVLSREDYEGLSLPGQP

AAN52483.1|Mus_musculus_castaneu ------MCSQEEFQAQRSQLVALLIS-GSLEGFESILDWLLSWDVLSREDYEGLSLPGQP

Q8K3Z0.1|Mus_musculus RSSCCDMCSQEEFQAQRSQLVALLIS-GSLEGFESILDWLLSWDVLSREDYEGLSLPGQP

AAN52480.1|Mus_musculus_musculus ------MCSQEEFQAQRSQLVALLIS-GSLEGFESILDWLLSWDVLSREDYEGLSLPGQP

EHB15612.1|Heterocephalus_glaber SPEGHEMNSQEAFQAQRSQLVGLLVS-GSLEGFERILDWLLCWEVLSWEEYEGLCLPGQP

KFO37911.1|Fukomys_damarensis MLRGREMNSREAFQAQRSQLVGLLVS-GSLEGFESILDWLLSWEVLSWEEYEGLRLLGQP

BAH24204.1|Sus_scrofa ------MCAQEAFQAQRSQLVELLVS-GSLEGFESILDWLLSWDVLSWEDYEGLSLLGQP

AHL44890.1|Capra_hircus ------MCAQDAFQTQRSQLVELLVS-GSLESFESIVDRLLSREVLSWEDYEGLSLVGQP

AJC01046.1|Bubalus_bubalis ------MCAQDVFQTQRSQLVELLVS-GSLESFESILDRLLSREVLSWEDYEGLSLVGQP

ELR51388.1|Bos_mutus --PDCEMCAQDAFQTQRSQLVELLVS-GSLEGFESILDRLLSREVLSWEDYEGLSLVGQP

AAS09828.1|Bison_bison ------MCAQDAFQTQRSQLLELLVS-GSLEGFESILDRLLSREVLSWEDYEGLSLVGQP

AAS09827.1|Bos_indicus ------MCAQDAFQTQRSQLVELLVS-GSLEGFESILDRLLSREVLSWEDYEGLSLVGQP

NP_001002889.1|Bos_taurus ------MCAQDAFQTQRSQLVELLVS-GSLEGFESILDRLLSREVLSWEDYEGLSLVGQP

NP_001273968.1|Canis_lupus_famil ------MCTQEAFQTQRSQLVGLLVS-GSLEGFESILDWLLSWEVLSWEDYEGLSLLGQP

ELK15377.1|Pteropus_alecto ------MCAQEAFQAQRSQLVGLLVS-GSLEGFESVLDWLLSWDVLSWEDYEGLSVLGQP

AAS89989.1|Saguinus_oedipus ------MCSQEAFQAQRSQLVELLVS-GSLEGFESVLDWLLSWEVLSWEDYEGFHLLGQP

Q53B88.1|Hylobates_lar QYLGCEMCSQEAFQAQRSQLVELLVS-GSLEGFESVLDWLLSWEVLSWEDYEGFHLLGQP

NP_071445.1|Homo_sapiens 22 HSPGCEMCSQEAFQAQRSQLVELLVS-GSLEGFESVLDWLLSWEVLSWEDYEGFHLLGQP 80

NP_001098710.1|Pan_troglodytes HSLGCEMCSQEAFQAQRSQLVELLVS-GSLEGFESVLDWLLSWEVLSWEDYEGFHLLGQP

AAY97879.1|Danio_rerio ----------------RAELLAVLWGGGSDEPLESVLDLLLAQEVLVWEDYLRVRVAEKP

ACX71753.1|Ctenopharyngodon_idel ------MSAHQLIITQRAELLTVLCGGGSAEPLECVLDLLLAWEVLVWEDYLNIRVAEKP

AFY26969.1|Carassius_auratus ------MTAHQLILKQRAELLAALCGGGNAEPLDCVLDLLLACEVLVWEDYLSIRVAEKP

AEG89706.1|Labeo_rohita ------MSAHQLVLKQRAELLSVLCGGGSAEPLDCVLDLLLAWEILVWEDYLSIRVAEKS

ADV31549.1|Oncorhynchus_mykiss ------MSARQLVLRQRAELLNALCCGGSSEGLESVSDLLLSWGMLFWEDYQNVLVPCRG

NP_001035913.1|Takifugu_rubripes ------MRVQDLVLNQRTEILRVLCSSGSAEPVEHVTDILLSQGELTWEDYQSIHVSGRP

AFV53358.1|Epinephelus_coioides ------MFVQELVLKQRTEILHALCSSGSAEHLEQVLDILLSQGELIWEDYQNVQVPGRA

AJF23836.1|Larimimichthys_crocea VVETLTMFAQEFVLKQRTEILHALCSSGSTEHLERVLDILLAQGELIWEDYQNI------

*:::: * *. * .: : * **. * .*:* .

ERE77543.1|Cricetulus_griseus LSHSARRLLDTVWNKGDWGCQKLLEAVQEAQANSHTLEL---HGC---W------DTHSI

NP_001099642.1|Rattus_norvegicus LSHSARRLLDTVCNKGVWGCQKLFEAVQEAQANSHTFEL---QGC---W------DVHSL

AAN52938.1|Mus_spretus LSHSARRLLDTVWNKGVWGCQKLLEAVQEAQANSHTFEL---HGS---W------DTHSL

AAN52482.1|Mus_musculus_domestic LSHSARRLLDTVWNKGVWGCQKLLEAVQEAQANSHTFEL---YGS---W------DTHSL

AAN52483.1|Mus_musculus_castaneu LSHSARRLLDTVWNKGVWGCQKLLEAMQEAQANSHTFEL---HGS---W------DTHSL

Q8K3Z0.1|Mus_musculus LSHSARRLLDTVWNKGVWGCQKLLEAVQEAQANSHTFEL---YGS---W------DTHSL

AAN52480.1|Mus_musculus_musculus LSHSARRLLDTVWNKGVWGCQKLLEAVQEAQANSHTFEL---YGS---W------DTHSL

EHB15612.1|Heterocephalus_glaber LSHVARRLLDTVWNKGSWGCQKLFEAVQEAQADSQPPEL---HNS---W------DPHSG

KFO37911.1|Fukomys_damarensis LSQVARRLLDTIWNKGSWGCQKFFAAVQQAQADSQSPEQ---HDS---W------DLHSG

BAH24204.1|Sus_scrofa LSHLARRLLDTVWSKGTWGCEQLVAAVREAQADSQPPEL---PGC---W------NPHSP

AHL44890.1|Capra_hircus ISHLARRLLDTIWNKGAWGCEQLTAAVWEAQADSQPPEL---PSS---W------DPHSP

AJC01046.1|Bubalus_bubalis ISHLARRLLDTIWNKGAWGCEQLTAAVREAQADSQPPEL---PSS---W------DPHSP

ELR51388.1|Bos_mutus ISHLARRLLDTIWNKGAWGCEQLTAAVREAQADSQPPEL---PSS---W------DPHSP

AAS09828.1|Bison_bison ISHLARRLLDTIWNKGAWGCEQLTAAVREAQADSQPPEL---PSS---W------DPHSP

AAS09827.1|Bos_indicus ISHLARRLLDTIWNKGTWGCEQLTAAVREAQADSQPPEL---PSS---W------DPHSP

NP_001002889.1|Bos_taurus ISHLARRLLDTIWNKGTWGCEQLTAAVREAQADSQPPEL---PSS---W------DPHSP

NP_001273968.1|Canis_lupus_famil LSYLARRLLDTVWNKGTWGCEQLVAAVREAQTDCQALEL---SSC---W------DPHSP

ELK15377.1|Pteropus_alecto LSHLARRLLDTVWNKGAWACEQLLAAVQEARADSQSPRL---HGH---W------DPHSS

AAS89989.1|Saguinus_oedipus LSHLARRLLDTVWNKGTWACQKLIAAAQEAQADSQSPKL---HGC---W------DPHSL

Q53B88.1|Hylobates_lar LSHLARRLLDTVWNKGTWACQKLIAAAQEAQADSQSPKL---HGC---W------DPHSL

NP_071445.1|Homo_sapiens 81 LSHLARRLLDTVWNKGTWACQKLIAAAQEAQADSQSPKL---HGC---W------DPHSL 128

NP_001098710.1|Pan_troglodytes LSHLARRLLDTVWNKGTWACQKLIAAAQEAQADSQSPKL---HGC---W------DPHSL

AAY97879.1|Danio_rerio LCANIRQLLDLVYDKGEDACSYFLAAIEQELAEEQKAGLCFGNGC---VM---VGKDRPA

ACX71753.1|Ctenopharyngodon_idel LCSNIRQLLDVVHDKGEDACSLFLAAINQVLPEEQKAGLCFGKGC---AV---VGKNRPD

AFY26969.1|Carassius_auratus LCSNVRHLLDVVYDKGEDACSLFLAAMNQVVPEEQKAGLCFGKEC---AV---VEKNRPD

AEG89706.1|Labeo_rohita LCSNVRQLLDVVYDKGEDACSLLLAAMNQVLPEEQKAGLSFGKEC---AV---VDKNRPD

ADV31549.1|Oncorhynchus_mykiss LCANTRVLLDLVYTKGDETCRLLLAAFKQVLPEAQRAGLSF-GNCRTADLENTRDDTREL

NP_001035913.1|Takifugu_rubripes LYTNARQLLDVVYLKGESSCGLFLEALKQVLPEEQPAGFSF-PDH---WWDPEVREECQN

AFV53358.1|Epinephelus_coioides LYTNARQLLDLVRAKGVDTCELFLAALKQVLPEAQRAGLSF-SGC---CLNLEEKKEYQS

AJF23836.1|Larimimichthys_crocea ----------------------------QVLPESQIVGLSF-AGC---NSNLEEKEEYQS

: .: : .

ERE77543.1|Cricetulus_griseus QPT--RDLQSHRPAIVRGLYNHVEAMLELAREQGFLSQYECDEIRLPIFTSSQRARRLLD

NP_001099642.1|Rattus_norvegicus HPT--RDLQSHRPAIVRRLCSHVEAILELARDEGFLSQYECEEIRLPIFTSSQRARRLLD

AAN52938.1|Mus_spretus HPT--RDLQSHRPAIVRRLYNHVEAMLELARERGFLSQYECEEIRLPIFTSSQRARRLLD

AAN52482.1|Mus_musculus_domestic HPT--RDLQSHRPAIVRRLYNHVEAMLELAREGGFLSQYECEEIRLPIFTSSQRARRLLD

AAN52483.1|Mus_musculus_castaneu HPT--RDLQSHRPAIVRRLYNHVEAMLELAREGGFLSQYECEEIRLPIFTSSQRARRLLD

Q8K3Z0.1|Mus_musculus HPT--RDLQSHRPAIVRRLYNHVEAMLELAREGGFLSQYECEEIRLPIFTSSQRARRLLD

AAN52480.1|Mus_musculus_musculus HPT--RDLQSHRPAIVRRLYNHVEAMLELAREGGFLSQYECEEIRLPIFTSSQRARRLLD

EHB15612.1|Heterocephalus_glaber HPA--QDLLNHRPAIVRKLYGHVEDVLAVARERGFISQYECDEIRLPIFTASQRARRLLD

KFO37911.1|Fukomys_damarensis HPA--RDLLNHRPAIVRKLYSHVEDVLAVARERGFISQYECDEIRLPIFTASQRARRLLD

BAH24204.1|Sus_scrofa HPA--RDLQSHRPAVVRRLYSHVDGVLERTQEQGFISRYECDEIRRPIFTSCQRARRLLD

AHL44890.1|Capra_hircus HPA--RDLQSHRPAIVRRLYGHVEGVLDLTQQRGFISQYETDEIRRPIFTSSQRARRLLD

AJC01046.1|Bubalus_bubalis HPA--RDLQSHRPAIVRRLYGHVEGVLDLTQQRGFISQYETDEIRRPIFTSSQRARRLLD

ELR51388.1|Bos_mutus HPA--RDLQSHRPAIVRRLYGHVEGVLDLTQQRGFISQYETDEIRRPIFTSSQRARRLLD

AAS09828.1|Bison_bison HPA--RDLQSHRPAIVRRLYGHVEGVLDLTQQRGFISQYETDEIRRPIFTSSQRARRLLD

AAS09827.1|Bos_indicus HPA--RDLQSHRPAIVRRLYGHVEGVLDLTQQRGFISQYETDEIRRPIFTSSQRARRLLD

NP_001002889.1|Bos_taurus HPA--RDLQSHRPAIVRRLYGHVEGVLDLTQQRGFISQYETDEIRRPIFTSSQRARRLLD

NP_001273968.1|Canis_lupus_famil HPA--RDLQSHRPAIVRRLYSHVEDVLNLAWEQGFISQYERDEIRLPIFTSSQRARRLLD

ELK15377.1|Pteropus_alecto HPV--RDLRSHRPAIVRRLYSHVEGVLDRAQERGFVSPYECDEIRLPVFTSSQRARRLLD

AAS89989.1|Saguinus_oedipus HPA--RDLQSHRPAIVRRLHSHVENMLDLAWERGFVSQYECDEIRLPIFTPSQRARRLLD

Q53B88.1|Hylobates_lar HPA--RDLQSHRPAIVRRLHSHVEGVLDLAWERGFVSQYECDEIRLPIFTPSQRARRLLD

NP_071445.1|Homo_sapiens 129 HPA--RDLQSHRPAIVRRLHSHVENMLDLAWERGFVSQYECDEIRLPIFTPSQRARRLLD 186

NP_001098710.1|Pan_troglodytes HPA--RDLQSHRPAIVRRLHNHVENMLDLAWERGFVSQYECDEIRLPIFTPSQRARRLLD

AAY97879.1|Danio_rerio TAT--STLLADRPMLVRRLRDNIDGALNILLTTGCFSIKDCDSVQLPVYTPSQQVRRLLD

ACX71753.1|Ctenopharyngodon_idel TAT--LMLLVDRPVLVTKLRDNIDGALNVLLTTGCFTIQDCDGVQLPVYTPSQQVRRLLD

AFY26969.1|Carassius_auratus TAT--LTLLADRPVLVRKLRDNIDGALNALLTTGCFTIKDCDAVQLPVYTPSQQVRRLLD

AEG89706.1|Labeo_rohita TAA--SMLLADRPVLVGKLRDNIDGALNALLTTGCFTIKDCDAVQLPVYTPSQQVRRLLD

ADV31549.1|Oncorhynchus_mykiss HGTAIQTLQTDRPDLVRRLRDHIEGALEVLLHTGSFTPSDCNEVQLPVYTPSQQARHLLD

NP_001035913.1|Takifugu_rubripes TFC--QTLLTQRPSLVSTLQHCVDGALEALLTSAHFTSADCEGIRLPLRTPSQQARCLLD

AFV53358.1|Epinephelus_coioides TST--QTLLTQRPSLVNKLQGCIDGALNALVTSEYFTSADCDEVRLPIHTPSQQARRLLD

AJF23836.1|Larimimichthys_crocea TST--HTLLTQRPSLVSKLLGRIHGALEALVISGHFTSADCDEVRLPVYTPSQQARRLLD

* ** :* * : * .: : : :. *: *..*..* ***

ERE77543.1|Cricetulus_griseus LATVKANGLAAFLLQHIKELPSP-SLPYEA-----AECQRFASKLRTMVSAQSRFLSTYD

NP_001099642.1|Rattus_norvegicus LAAVKANGLAAFLLQHVRKLPASLSLPYEA-----AECQKFTAKLRTMVSAQSRFLSTYD

AAN52938.1|Mus_spretus LAAVKANGLAAFLLQHVRELPAPLPLPYEA-----AECQKFIPKLRTMVLAQSRFLSTYD

AAN52482.1|Mus_musculus_domestic LAAVKANGLAAFLLQHVRELPAPLPLPYEA-----AECQKFISKLRTMVLAQSRFLSTYD

AAN52483.1|Mus_musculus_castaneu LAAVKANGLAAFLLQHIRELPAPLPLPYEA-----AECQKFISKLRTMVLAQSRFLSTYD

Q8K3Z0.1|Mus_musculus LAAVKANGLAAFLLQHVRELPAPLPLPYEA-----AECQKFISKLRTMVLTQSRFLSTYD

AAN52480.1|Mus_musculus_musculus LAAVKANGLAAFLLQHVRELPAPLPLPYEA-----AECQKFISKLRTMVLAQSRFLSTYD

EHB15612.1|Heterocephalus_glaber LATVKANGLAAFLLQHIQESPALLSLPFEA-----AECQKYISKLRTTVSAQSRSLSTYD

KFO37911.1|Fukomys_damarensis LATVKTNGLAAFLLQHLQESPALLSLPFEA-----AECQKYISKLRATVSAQSRSLSTYD

BAH24204.1|Sus_scrofa LAAVKANGLAAFLLQCVQELPIPVALPFED-----AACKKYMSKLRTTVSAQSRFLSTYD

AHL44890.1|Capra_hircus LAAVKANGLAAFLLQCIQELPVPLALPFED-----AACKKYMSKLRTVISAQSRFLSTYD

AJC01046.1|Bubalus_bubalis LATVKANGLAAFLLQCIQELPVPLALPFED-----AACKKYVSKLRTVISAQSRFLSTYD

ELR51388.1|Bos_mutus LATVKANGLAAFLLQCIQELPVPLALPFED-----AACKKYVSKLRTVISAQSRFLSTYD

AAS09828.1|Bison_bison LATVKANGLAAFLLQCIQELPVPLALPFED-----AACKKYVSKLRTVISAQSRFLSTYD

AAS09827.1|Bos_indicus LATVKANGLAAFLLQCIQELPVPLALPFED-----AACKKYMSKLRTVISAQSRFLSTYD

NP_001002889.1|Bos_taurus LATVKANGLAAFLLQCIQELPVPLALPFED-----AACKKYVSKLRTVISAQSRFLSTYD

NP_001273968.1|Canis_lupus_famil LATVKANGLAAFLLRHVQELPVPLAMPSED-----AACKKYMSKLRTTISSQSRFLSTYD

ELK15377.1|Pteropus_alecto LATVKVNGLAAFLLQHVQELPVPTTLPFED-----AACTKYMAKLRATVSAQSRFLSTYD

AAS89989.1|Saguinus_oedipus LATVKANGLAAFLLQHVQELPVPLALPLEA-----ATCKKYMAKLRTTVSAQSRFLSTYD

Q53B88.1|Hylobates_lar LATVKANGLAAFLLQHVQELPVPLALPLEA-----ATCRKYMAKLRTTVSAQSRFLSTYD

NP_071445.1|Homo_sapiens 187 LATVKANGLAAFLLQHVQELPVPLALPLEA-----ATCKKYMAKLRTTVSAQSRFLSTYD 241

NP_001098710.1|Pan_troglodytes LATVKANGLAAFLLQHVQELPVPLALPLEA-----ATCKKYMAKLRTTVSAQSRFLSTYD

AAY97879.1|Danio_rerio QVKFKGETAAKTLLEYLEQPEPTSPISAEKENTPSADCLVY-KKLRSSVASQSLFLSTYG

ACX71753.1|Ctenopharyngodon_idel QVKFKGETAAKTLLQYLEQTEPSKPKPEDKENHLSADCLLYQKKLRSSVAAQSLFLSTYG

AFY26969.1|Carassius_auratus QVRFKGETAAKTLLQYLEQTETSRPNPEDKENHLSANCMLYQKKLRSSVAAQSHSLNTYG

AEG89706.1|Labeo_rohita QVRFKGETAAKTILQYLEQTEPTRPNPEDKENHLSANVLLYQKKLRSSVAAQSHSLSTYG

ADV31549.1|Oncorhynchus_mykiss QVRSKGEPAAKVLLQYFQQTEDHPSPSNQG--APPKECLSYQKKLRSTASAQSHFLGTYG

NP_001035913.1|Takifugu_rubripes HVRAKGEAAAKFLLEYIQQGEESGSILNKKTQTVSREFLKFQKKLRSSLSAQSCFLSTYG

AFV53358.1|Epinephelus_coioides HVRSKGESAAEVVLQYIQQTQASGSPANQETWTPPKEVLKYQKKLSSSVSAQSCFLSTYG

AJF23836.1|Larimimichthys_crocea HVRPKGELAAKVVLQYIQQKQETGSALNPEKLTPPKEFLKYQKKLSSSVSAQSCFLSTYG

. * : * :* . : . : ** : :** *.**.

ERE77543.1|Cricetulus_griseus GSENLCLEDIYTENILELRTEVGMAGALQKSPAILGLEELFGTHGHLNKDADTVLVVGEA

NP_001099642.1|Rattus_norvegicus GSENLCLEDIYTENTLELRTEVGTAGALQKSPATLGLEELFGTHGHLNKDADTILVVGEA

AAN52938.1|Mus_spretus GSENLCLEDIYTENILELRTDVGTAGALQKSPAILGLEDLFDTHGHLNRDADTILVVGEA

AAN52482.1|Mus_musculus_domestic GSENLCLEDIYTENILELRTEVGTAGALQKSPAILGLEDLFDTHGHLNRDADTILVVGEA

AAN52483.1|Mus_musculus_castaneu GSENLCLEDIYTENILELRTEVGTAGALQKSPAILGLEDLFDTHGHLNRDADTILVVGEA

Q8K3Z0.1|Mus_musculus GSENLCLEDIYTENILELQTEVGTAGALQKSPAILGLEDLFDTHGHLNRDADTILVVGEA

AAN52480.1|Mus_musculus_musculus GSENLCLEDIYTENILELQTEVGTAGALQKSPAILGLEDLFDTHGHLNRDADTILVVGEA

EHB15612.1|Heterocephalus_glaber GVENLCLEDIYTENILELRTEVGMVGAWKKSPANLGLEELFSTQGHLNEDADTVLLVGEA

KFO37911.1|Fukomys_damarensis GAETLRLEDIYTENILELRMEVGMVGARKKSPATLGLEELFSTQGHLNEDADTVLVVGEA

BAH24204.1|Sus_scrofa GAENLCLEEIYTENVLEVRTEGGMTGPPQQSPATLSLGELFSPQGHLNKDADTVLVVGEA

AHL44890.1|Capra_hircus GAENLCLEEVYTENVLEIQMEVGMAGPSQQSPTTLGLEELFSTRDHFNKEADTVLVVGEA

AJC01046.1|Bubalus_bubalis GAENLCLEEVYTENVLEIQMEVGMAGPSQQSPTTLGLEELFSTRDHFNKEADTVLVVGEA

ELR51388.1|Bos_mutus GAENLCLEEVYTENVLEIQMEVGMAGPSQQSPTTLGLEELFSTCDHFNKEADTVLVVGEA

AAS09828.1|Bison_bison GAENLCLEEVYTENVLEIQMEVGMAGPSQQSPTTLGLEELFSTRDHFNKEADTVLVVGEA

AAS09827.1|Bos_indicus GAENLCLEEVYTENVLEIQMEVGMAGPSQQSPTTLGLEELFSTRDHFNKEADTVLVVGEA

NP_001002889.1|Bos_taurus GAENLCLEEVYTENVLEIQMEVGMAGPSQQSPTTLGLEELFSTRDHFNKEADTVLVVGEA

NP_001273968.1|Canis_lupus_famil GAENLCLEEIYTENVLEIRTEMGLARSPQKSPATLSLEELFSTCGHLNEDADTVLVVGEA

ELK15377.1|Pteropus_alecto GAENLCLEEIYTENVLEIRTEVGTAEPLQKSPATLGLEDLFSTRGHLNEDADTVLVVGEA

AAS89989.1|Saguinus_oedipus GAETLCLEDIYTENVLEVWADAGMAGPPQKSPATLGLEELLSTPGHLSEDADTVLVVGEA

Q53B88.1|Hylobates_lar GAETLCLEDIYTENVLEVWADVGTAGPPPKSPATLGLEELFSTPGHLNDDADTVLVVGEA

NP_071445.1|Homo_sapiens 242 GAETLCLEDIYTENVLEVWADVGMAGPPQKSPATLGLEELFSTPGHLNDDADTVLVVGEA 301

NP_001098710.1|Pan_troglodytes GAETLCLEDIYTENVLEVWADVGMAGPPQKSPATLGLEELFSTPGHLNDDADTVLVVGEA

AAY97879.1|Danio_rerio GTGRFSLDDIYTDGHLEV---MNSSGET----TTLGLEDVVGPMGTLNEDADTVLVSGEA

ACX71753.1|Ctenopharyngodon_idel GAGRFSLDDIYTDGLLEV---VDGSGET----TTLGLEDMVGPIGTLNEDADTILLSGEA

AFY26969.1|Carassius_auratus GTGHFSLDDIYTDGLLEV---MDGSGET----TTLGLEDVVGPLGTLNDDADTFLVSGEA

AEG89706.1|Labeo_rohita GTGRFSLDDIYTDGLLEV---MNGSGET----TTLGLEDVVGPLGTLNEDADTVLVSGEA

ADV31549.1|Oncorhynchus_mykiss GTSSLSLEDIYTEGQLEL---AQGDSEAQPQTGALGLEDVVGLVGTLNQEADTVLVSGEA

NP_001035913.1|Takifugu_rubripes GTSHMSLDDIYTDGQLEL---AEACPDVH---GPIGLKDITGEVGTINEEADTVLVSGEA

AFV53358.1|Epinephelus_coioides GTSHMSLDDIYTEGQLEL---GHNTADVH---GSLGLEDVVGSVGTVNEEADTVLVSGEA

AJF23836.1|Larimimichthys_crocea GTSHMSLDDIYTEGQLEL---AQYCADVH---GPLGLEDIVGTVGTVNEEADTVLVSGEA

* : *:::**:. **: :.* :: . . .. :***.*: ***

ERE77543.1|Cricetulus_griseus GSGKSTLLQRLLLLWATGQNFQEFLFVFPFSCRQLQCMAKPLSLKTLLFEHCCWPDVGQH

NP_001099642.1|Rattus_norvegicus GSGKSTLLQRLHLLWASGQNFREFLFIFPFSCRQLQCMTKPLSLRTLLFEHCCWPDVGQD

AAN52938.1|Mus_spretus GSGKSTLLQRLHLLWATGRSFQEFLFIFPFSCRQLQCVAKPLCLRTLLFEHCCWPDVAQD

AAN52482.1|Mus_musculus_domestic GSGKSTLLQRLHLLWATGRSFQEFLFIFPFSCRQLQCVAKLLSLRTLLFEHCCWPDVAQD

AAN52483.1|Mus_musculus_castaneu GSGKSTLLQRLHLLWATGRSFQEFLFIFPFSCRQLQCVAKPLSLRTLLFEHCCWPDVAQD

Q8K3Z0.1|Mus_musculus GSGKSTLLQRLHLLWATGRSFQEFLFIFPFSCRQLQCVAKPLSLRTLLFEHCCWPDVAQD

AAN52480.1|Mus_musculus_musculus GSGKSTLLQRLHLLWATGRSFQEFLFIFPFSCRQLQCVAKPLSLRTLLFEHCCWPDVAQD

EHB15612.1|Heterocephalus_glaber GSGKSTLLQRLHLLWATGQDFQEFLFVFPFSCRQLQCVAKPLSVRTLLFEYCCWPDAGQQ

KFO37911.1|Fukomys_damarensis GSGKSTLLQRLHLLWATGQDFQEFLFVFPFSCRQLQCVAKPLSVRTLLFEHCCWPDVGQQ

BAH24204.1|Sus_scrofa GSGKSTLLQQVHLLWASGQAFQEFLFVFPFSCRQLQCLAKSLSLQTLLFEHCCWPDRGQQ

AHL44890.1|Capra_hircus GSGKSTLLQQLHLLWASGRAFQEFLFVFPFSCRQLQCLVKPLSVQTLLFEHCCWPDLGPQ

AJC01046.1|Bubalus_bubalis GSGKSTLLQQLHLLWASGRAFQEFLFVFPFSCRQLQCLVKPLSVQTLLFEHCCWPDLGPQ

ELR51388.1|Bos_mutus GSGKSTLLQQLHLLWASGRAFQEFLFVFPFSCRQLQCLVKPLSMRTLLFEHCCWPDLGPQ

AAS09828.1|Bison_bison GSGKSTLLQQLHLLWASGRAFQEFLFVFPFSCRQLQCLVKPLSMRTLLFEHCCWPDLGPQ

AAS09827.1|Bos_indicus GSGKSTLLQQLHLLWASGRAFQEFLFVFPFSCRQLQCLVKPLSMRTLLFEHCCWPDLGPQ

NP_001002889.1|Bos_taurus GSGKSTLLQQLHLLWASGRAFQEFLFVFPFSCRQLQCLVKPLSMRTLLFEHCCWPDLGPQ

NP_001273968.1|Canis_lupus_famil GSGKSTLLQRMHLLWASGRDFQEFLFVFPFSCRQLQCVAKPLSVQMLLFEHCCWPDFGQQ

ELK15377.1|Pteropus_alecto GSGKSTLLQQLHLLWATGRDFQEFLFVFPFSCRQLQCIAKPLSVRTLLFEHCCWPDLGQQ

AAS89989.1|Saguinus_oedipus GSGKSTLLQRLHFLWAAGRDFQEFLFVFPFSCRQLQCVAKPLSVRMLLFEHCCWPDVDQQ

Q53B88.1|Hylobates_lar GSGKSTLLQRLHLLWAAGRDFQEFLFVFPFSCRQLQCMAKPLSVRTLLFEHCCWPDVGQE

NP_071445.1|Homo_sapiens 311 GSGKSTLLQRLHLLWAAGQDFQEFLFVFPFSCRQLQCMAKPLSVRTLLFEHCCWPDVGQE 361

NP_001098710.1|Pan_troglodytes GSGKSTLLQRLHLLWAAGRDFQEFLFVFPFSCRQLQCMAKPLSVRTLLFEHCCWPDVGQE

AAY97879.1|Danio_rerio GSGKSTLVQRLHLLWAREALLLNTFLLFPFSCRKLNAEHRELSLKELLFLHCCWPDRNQD

ACX71753.1|Ctenopharyngodon_idel GSGKSTLLQRLHLLWAREALLTNTFLLFPFSCRKLNAEHRELSLKELLFLHCCWPDRSQD

AFY26969.1|Carassius_auratus GSGKSTLLQRLHLLWAREALLQNIFLLFPFSCRKLNAEHRELSLKELLFLHCCWPDRGQD

AEG89706.1|Labeo_rohita GSGKSTLLQRLHLLWAREALLTNTFLLFPFSCRKLNAEHRELSLKELLFLHCCWPDRGQD

ADV31549.1|Oncorhynchus_mykiss GSGKSTLLQRLHLLWARGAALHDFFLLFSFSCRRLSSEQRELSLRELLFLHCCWPDGDQD

NP_001035913.1|Takifugu_rubripes GSGKSTLLQRLHLLWAREAALLEYLLLFPFSCRRLNTELSELSFKELLFQHCCWPDRDQD

AFV53358.1|Epinephelus_coioides GVGKSTLLQRLHLLWARGAAFQDFLLLFPFSCRRLNSEHKEMSVQELLFQHCCWPDREQE

AJF23836.1|Larimimichthys_crocea GSGKTTLLQRLHLLWARGVALQQFLLLFPFSCRRLNSEHRELSVKELLFQHCCWPDRDQE

* **:**:*.: :*** : : :::*.****.*. :... *** :*****

ERE77543.1|Cricetulus_griseus DVFQFLLDHPDRVLLTFDGLDEFKFRFTD-RERHCSPTDPTSVQTLLFNLLQGNLLKNAH

NP_001099642.1|Rattus_norvegicus DVFQFLLDHPDRVLLTFDGLDEFKFRFTD-RERHCSPLDPTSVQTLLFNLLQGNLLKNAC

AAN52938.1|Mus_spretus DVFQFLLDHPDRVLLTFDGLDEFKFRFTD-RERHCSPIDPTSVQTLLFNLLQGNLLKNAC

AAN52482.1|Mus_musculus_domestic DVFQFLLDHPDRVLLTFDGLDEFKFRFTD-RERHCSPIDPTSVQTLLFNLLQGNLLKNAC

AAN52483.1|Mus_musculus_castaneu DVFQFLLDHPDRVLLTFDGLDEFKFRFTD-RERHCSPIDPTSVQTLLFNLLQGNLLKNAC

Q8K3Z0.1|Mus_musculus DVFQFLLDHPDRVLLTFDGLDEFKFRFTD-RERHCSPIDPTSVQTLLFNLLQGNLLKNAC

AAN52480.1|Mus_musculus_musculus DVFQFLLDHPDRVLLTFDGLDEFKFRFTD-RERHCSPIDPTSVQTLLFNLLQGNLLKNAC

EHB15612.1|Heterocephalus_glaber DVFQFLLDHPDRVLLTFDGFDEFRFKFTD-QERHCSPTDPTSVQTLLFNLLQGNLLKNAR

KFO37911.1|Fukomys_damarensis VVFEFLLDHPDRVLLTFDGFDEFRFKFTD-QERHCSPTDPTSVQTLLFNLLQGNLLKNAR

BAH24204.1|Sus_scrofa DVFQVLLDHPERILLTFDGFDEFRFRFTD-HERHCCPTAPTSVQSLLFNLLQGNLLKNAR

AHL44890.1|Capra_hircus DIFQVLLDHPERILLTFDGFDEFRFRFTD-RERHCCPTAPTSVQSLLFNLLQGNLLKNAC

AJC01046.1|Bubalus_bubalis DVFQVLLDHPERILLTFDGFDEFRFRFTD-RERHCCPTAPTSVQSLLFNLLQGNLLKNAR

ELR51388.1|Bos_mutus DVFQVLLDHPERILLTFDGFDEFRFRFTD-QERHCCPTAPTSVQSLLFNLLQGNLLKNAR

AAS09828.1|Bison_bison DVFQVLLDHPERILLTFDGFDEFRFRFTD-QERHCCPTAPTSVQSLLFNLLQGNLLKNAR

AAS09827.1|Bos_indicus DVFQVLLDHPERILLTFDGFDEFRFRFTD-QERHCCPTAPTSVQSLLFNLLQGNLLKNAR

NP_001002889.1|Bos_taurus DVFQVLLDHPERILLTFDGFDEFRFRFTD-QERHCCPTAPTSVQSLLFNLLQGNLLKNAR

NP_001273968.1|Canis_lupus_famil EVFQFLLDHPNRVLLTFDGFDEFRFRFSD-HERHCSPTDPTSVQNLLFNLLQGNLLKNAR

ELK15377.1|Pteropus_alecto EVFQFLLDHPDRVLLTFDGFDEFRFRFTD-RERHCSPTDPMSVQNLLFNLLQGNLLKNAR

AAS89989.1|Saguinus_oedipus DIFQFLLDHPDRVLLTFDGFDEFKFRFSD-RERHCSPTDPTSVQTLLFNLLQGNLLKNAR

Q53B88.1|Hylobates_lar DIFQLLLDHPDRVLLTFDGFDEFKFRFTD-RERHCSPTDPTSVQTLLFNLLQGNLLKNAR

NP_071445.1|Homo_sapiens 362 DIFQLLLDHPDRVLLTFDGFDEFKFRFTD-RERHCSPTDPTSVQTLLFNLLQGNLLKNAR 420

NP_001098710.1|Pan_troglodytes DIFQLLLDHPDRVLLTFDGFDEFKFRFTD-RERHCSPTDPTSVQTLLFNLLQGNLLKNAR

AAY97879.1|Danio_rerio EVFQFILDHPHLVLFTFDGLDEFKLGFTD-EERHCCPTKQVPIPVLLFNLLQGTLMKGVM

ACX71753.1|Ctenopharyngodon_idel EVFQFILDHPHMVLFTFDGLDEFKHGFRD-EERHCCPTKPVPIPVLLFNLLQGTLMKGIL

AFY26969.1|Carassius_auratus EVFQFILDHPNLVLFTFDGLDEFKQGFTD-EERHCCPTKQVPVPVLLFNLLQGTLMKGVV

AEG89706.1|Labeo_rohita EVFQFVLDHPNLVLFTFDGLDEFKQGFTD-EERHCCPTKQVPIPVLLFNLLQGTLMKGVM

ADV31549.1|Oncorhynchus_mykiss RIFQFILDHPHLILFTFDGLDELMQSFSDSEQRHCCPTQCAPVPTLLFNLLQGSLMKGVR

NP_001035913.1|Takifugu_rubripes EIFDFIQDHPHLILFTFDGLDELKQSFSD-EHRLCCPTQRAPVHVLLFNLIQGSLLKGVR

AFV53358.1|Epinephelus_coioides EIFQFILDHPHLILFTFDGLDELKQSFSD-EHRLCCPTQRAPVHTLLFNLIQGSLMKGVR

AJF23836.1|Larimimichthys_crocea EIFQFILDHPHLVLFTFDGLDELKQSFSD-EKRLCCPTQPAPVHILLFNFIQGSLMKGVR

:*:.: *** :*:****:**: * * * *.* .: ****::**.*:*.

ERE77543.1|Cricetulus_griseus KVLTSRPDAVSALLRKFVRIECHLKGFSEEGIEQYLRKHHREPGVADRLIHLLQATSALH

NP_001099642.1|Rattus_norvegicus KVLTSRPDAVSALLRKFVRTECHLKGFSEEGIKLYLRKHHREPGVADRLIHLIQATSALH

AAN52938.1|Mus_spretus KVLTSRPDAVSALLRKFVRTECQLKGFSEEGIQLYLRKHHREPGVADRLIQLIQATSALH

AAN52482.1|Mus_musculus_domestic KVLTSRPDAVSALLRKFVRTECQLKGFSEEGIQLYLRKHHREPGVADRLIQLIQATSALH

AAN52483.1|Mus_musculus_castaneu KVLTSRPDAVSALLRKFVRTECQLKGFSEEGIQLYLRKHHREPGVADRLIQLIQATSALH

Q8K3Z0.1|Mus_musculus KVLTSRPDAVSALLRKFVRTELQLKGFSEEGIQLYLRKHHREPGVADRLIQLIQATSALH

AAN52480.1|Mus_musculus_musculus KVLTSRPDAVSALLRKFVRTELQLKGFSEEGIQLYLRKHHREPGVADRLIQLIQATSALH

EHB15612.1|Heterocephalus_glaber KVLTSRPDAVSACLRKYVRTECHLRGFSEEGIELYLRKCHQDPGVADRLIQLLRATSVLH

KFO37911.1|Fukomys_damarensis KVLTSRPDAVSACLRKYVRTECHLRGFSEEGIELYLRKCHQDPDVADHLIQLLRATSVLH

BAH24204.1|Sus_scrofa KVLTSRPDAVSASLRKHVRSELSLKGFSEEGIELYLRKCHREPGVADRLICLLRATSALH

AHL44890.1|Capra_hircus KVLTSRPGAVSASLRKHVRTELSLKGFSEEGIELYLRKRHREPGVADRLLCLLRATSALH

AJC01046.1|Bubalus_bubalis KVLTSRPGAVSASLRKHVRTELSLKGFSEEGIELYLRKRHREPGVADRLLCLLRATSALH

ELR51388.1|Bos_mutus KVLTSRPSAVSASLRKHVRTELSLKGFSEEGIELYLRKRHREPGVADRLLCLLRATSALH

AAS09828.1|Bison_bison KVLTSRPSAVSASLRKHVRTELSLKGFSEEGIELYLRKRHREPGVADRLLCLLRATSALH

AAS09827.1|Bos_indicus KVLTSRPSAVSASLRKHVRTELSLKGFSEEGIELYLRKRHREPGVADRLLCLLRATSALH

NP_001002889.1|Bos_taurus KVLTSRPSAVSASLRKHVRTELSLKGFSEEGIELYLRKRHREPGVADRLLCLLRATSALH

NP_001273968.1|Canis_lupus_famil KVLTSRPDAVSALLRKYLRLEINLKGFSEEGIELYLRKCHREPGVADRLIRLLKTTSALH

ELK15377.1|Pteropus_alecto KVLTSRPAAVSAFLRKYVRAELALKGFSEDGIELYLRKRHREPGVADRLIGLLKATSALH

AAS89989.1|Saguinus_oedipus KVVTSRPAAVSAFLRKYIRTEFILKGFSEKGIELYLRKRHREPGVADRLIRLLQATSALH

Q53B88.1|Hylobates_lar KVVTSRPAAVSAFLRKYIRTEFNLKGFSEQGIELYLRKRHREPGVADRLIRLLQATSALH

NP_071445.1|Homo_sapiens 421 KVVTSRPAAVSAFLRKYIRTEFNLKGFSEQGIELYLRKRHHEPGVADRLIRLLQETSALH 480

NP_001098710.1|Pan_troglodytes KVVTSRPAAVSAFLRKYIRTEFNLKGFSEQGIELYLRKRHREPGVADRLIRLLQATSALH

AAY97879.1|Danio_rerio KVVTSRPHAVGPSLKRYLRKEVLLKGFSPGGIDCFVKKHYSDPAMATRVIESVQGNTALL

ACX71753.1|Ctenopharyngodon_idel KVVTSRPQAVGPSLKRYLRKEVHLNGFSPSGINCFVKKHHSDPAMARRVIDSLQVNTVLL

AFY26969.1|Carassius_auratus KVVTSRPQAVGPSLKRYLHKEVLLKGFSPGGIDCFVKKHHSDPALARRVIESLHANSVLL

AEG89706.1|Labeo_rohita KVVTSRPQAVGPSLKRYLRKEVLLKGFSPGGIDCFVKKHHSDPAMARRVIESLQANMVLL

ADV31549.1|Oncorhynchus_mykiss KVVTSRPEAVGPTLKRYLRKEVLLKGFSPGGIDCFVRKHHRDPTVATRVLESLRSNTALL

NP_001035913.1|Takifugu_rubripes KVVTSRPSAVTPVLKKNLCKEVLLKGFSPSGIDCFVRKHHSDPTVAAKVLQSLHTNTTLL

AFV53358.1|Epinephelus_coioides KVVTSRPEAVGPVLKKHLRKEVFLKGFSPNGIDCFVKKHHKDSTVAAKVLESLQTNTALL

AJF23836.1|Larimimichthys_crocea KVVTSRPEAVGPMLKTHLCKEVLLKGFSPSGIDCFVRKHHSDNTVATKVLESLQTNTALL

**:**** ** . *. : * *.*** **. ::.* : : :* .:: :. . .*

ERE77543.1|Cricetulus_griseus GLCHLPVFSWMVSRCHQELLLQ--NGGFPTTSTDMYLLILQHFLLHASPPDSYPLSLGP-

NP_001099642.1|Rattus_norvegicus GLCHLPVFSWMVSRCHRELLLQ--NRGFPTTSTDMYLLILQHFLLHASPPDSFPLGLGP-

AAN52938.1|Mus_spretus GLCHLPVFSWMVSRCHRELLLQ--NRGFPTTSTDMYLLILQHFLLHASPPDSSPLSLGP-

AAN52482.1|Mus_musculus_domestic GLCHLPVFSWMVSRCHRELLLQ--NRVFPTTSTDMYLLILQHFLLHASPPDSSPFGLGP-

AAN52483.1|Mus_musculus_castaneu GLCHLPVFSWMVSRCHRELLLQ--NRGFPTTSTDMYLLILQHFLLHASPPDSSPLGLGP-

Q8K3Z0.1|Mus_musculus GLCHLPVFSWMVSRCHRELLLQ--NRGFPTTSTDMYLLILQHFLLHASPPDSSPLGLGP-

AAN52480.1|Mus_musculus_musculus GLCHLPVFSWMVSRCHRELLLQ--NRGFPTTSTDMYLLILQHFLLHASPPDSSPLGLGP-

EHB15612.1|Heterocephalus_glaber GLCHLPVFSWMVSKCHQELLLQ--NEGSPKTTTDMYLLILQHFLLHASPPDAISHGLGP-

KFO37911.1|Fukomys_damarensis GLCHLPVFSWMVSKCHQELLLQ--NEGSPKTTTDMYLLILQHFLLHASPPDAISHGLGP-

BAH24204.1|Sus_scrofa GLCHLPVFSWMVSKCHQELLLQ--GRGSPKTTTDMYLLILQHFLLRASPLDSAAQHLGP-

AHL44890.1|Capra_hircus GLCHLPVFSWMVSKCHEELLLQ--GRGTPKTTTDMYLLILRHFLLHASPPPLATHGPGP-

AJC01046.1|Bubalus_bubalis GLCHLPVFSWMVSKCHEELLLQ--GRGSPKTTTDMYLLILQHFLLHASPLPLATHGLGP-

ELR51388.1|Bos_mutus GLCHLPVFSWMVSKCHEELLLQ--GRGSPKTTTDMYLLILRHFLLHASPLPLATHGLGP-

AAS09828.1|Bison_bison GLCHLPVFSWMVSKCHEELLLQ--GRGSPETTTDMYLLILRHFLLHASPLPLATHGLGP-

AAS09827.1|Bos_indicus GLCHLPVFSWMVSKCHEELLLQ--GRGSPKTTTDMYLLILRHFLLHASPLPLANHGLGP-

NP_001002889.1|Bos_taurus GLCHLPVFSWMVSKCHEELLLQ--GRGSPKTTTDMYLLILRHFLLHASPLPLATHGLGP-

NP_001273968.1|Canis_lupus_famil GLCHLPVVSWMVSKCHQELLLH--GGGSPKTSTDMYLLILQHFLLHASPPDSVPHSLGS-

ELK15377.1|Pteropus_alecto GLCHLPVFSWMVSKCHQELLLQ--GGGSPKTTTDMYLLILQHFLLHAAPPDSARQGLGP-

AAS89989.1|Saguinus_oedipus GLCHLPVFSWMVSKCHQELLLQ--EGGSPKTTTDMYLLILQHFLLHAVPPDSACHILGP-

Q53B88.1|Hylobates_lar GLCHLPVFSWMVSKCHQELLLQ--EGGSPKTTTDMYLLILQHFLLHAIPPDSASQGLGP-

NP_071445.1|Homo_sapiens 481 GLCHLPVFSWMVSKCHQELLLQ--EGGSPKTTTDMYLLILQHFLLHATPPDSASQGLGP- 537

NP_001098710.1|Pan_troglodytes GLCHLPVFSWMVSKCHQELLLQ--EGGSPKTTTDMYLLILQHFLLHATPPDSASQGLGP-

AAY97879.1|Danio_rerio GLCHIPVFCWIVIKCYQELLAG--QDGIPQTITDVYLLVLQHFFQRKSSQPQS--GLGK-

ACX71753.1|Ctenopharyngodon_idel GLCHIPVFCWIVTKCHQELLGG--QDGIPQTITDVYLLALQHFFQRKSSLSQG--ALGK-

AFY26969.1|Carassius_auratus SLCHIPVFCWIVTKCHQELLGS--QDGIPQTITDVYLLVLQHFFQRKSYQHQG--VLGK-

AEG89706.1|Labeo_rohita SLCHIPVFCWIVTKCHQELLAG--QDSVPQTITDVYLLVLQHFFQRKSSQSQG--VLGK-

ADV31549.1|Oncorhynchus_mykiss GLCHIPVFCWIVSKCYKELLGCGEGEGSPQTITYVYLMILHHFFQRRASQRNT---MGI-

NP_001035913.1|Takifugu_rubripes GLCHSPVLCWIVSCCHKELLGC--GEGSPQTITDVYLTIVEHFFQHHSPLRST----GKI

AFV53358.1|Epinephelus_coioides GLCHSPVLCWIVSQCHKELLGC--GEGSPQTITDVYLMILQHFFQHHSSLKST---IGL-

AJF23836.1|Larimimichthys_crocea GLCHSPVLCWIVSQCHKELLGC--GEGSPQTITDVYLMILQHFFQHQSSLKST---VGL-

.*** **..*:* *: *** * * * :** : **: . *

ERE77543.1|Cricetulus_griseus GLLQSRLSTLLHLGHLALQGLALSCYVFSAQQLQEAQVDADDISLGFLVRAQNV-VPGSK

NP_001099642.1|Rattus_norvegicus GLLQSRLSTLLHLGHLALQGLAMSCYVFSAQQLQAAQVDSEDISLGFLVRAQSV-VPGSK

AAN52938.1|Mus_spretus GLLQSRLSTLLHLGHLALRGLAMSCYVFSAQQLQAAQVDSDDISLGFLVRAQSS-GPGSK

AAN52482.1|Mus_musculus_domestic GLLQSRLSTLLHLGHLALRGLAMSCYVFSAQQLQAAQVDSDDISLGFLVRAQSS-VPGSK

AAN52483.1|Mus_musculus_castaneu GLLQSRLSTLLHLGHLALRGLAMSCYVFSAQQLQAAQVDSDDISLGFLVRAQSS-VPGSK

Q8K3Z0.1|Mus_musculus GLLQSRLSTLLHLGHLALRGLAMSCYVFSAQQLQAAQVDSDDISLGFLVRAQSS-VPGSK

AAN52480.1|Mus_musculus_musculus GLLQSRLSTLLHLGHLALRGLAMSCYVFSAQQLQAAQVDSDDISLGFLVRAQSS-VPGSK

EHB15612.1|Heterocephalus_glaber GLLQGKLPSLLHLGYLAFWGLGRCCYVFSAQQLQMAQLDPEDISLGFLVRAQGV-VPGSM

KFO37911.1|Fukomys_damarensis GLLQGKLPSLLHLGHLAFWGLGSCCYVFSAQQLQMAQVDAEDISLGFLVRAQGV-VPGSA

BAH24204.1|Sus_scrofa DLLRGSLPTLLHLGHLALWGLGTCCYVFSAEQLQAAHVDSEAVSLGFLVRAKSV-VPGGT

AHL44890.1|Capra_hircus SLIQRRLPTLLHLGHLALWGLGTCCYVFSAKQLQAAHVDSEDLSLGFLVHAKRV-VPGST

AJC01046.1|Bubalus_bubalis SLIQGRLPTLLHLGRLALWGLGTCCYVFSAKQLQAAHVDSEDLSLGFLVLAKRV-VPXST

ELR51388.1|Bos_mutus SLIQGRLPTLLHLGRLALWGLGTCCYVFSAKQLQAAHVDSEDLSLGFLVLAKRV-VPGST

AAS09828.1|Bison_bison SLIQGRLPTLLHLGRLALWGLGTCCYVFSAKQLQAAHVDSEDLSLGFLVLAKRV-VPGST

AAS09827.1|Bos_indicus SLIQGRLPTLLHLGRLALWGLGTCCYVFSAKQLQAAHVDSEDLSLGFLVLAKRV-VPGST

NP_001002889.1|Bos_taurus SLIQGRLPTLLHLGRLALWGLGTCCYVFSAKQLQAAHVDSEDLSLGFLVLAKRV-VPGST

NP_001273968.1|Canis_lupus_famil HLLRGRLPTLLHLGWLALWGLGMCCYVFSAKQLQAAHIDDEDISLGFLVHAKTV-GPGST

ELK15377.1|Pteropus_alecto SLLRGRLPTLLHLGRLALWGLGMSCYVFSAPQLQAAHVDSEDVSLGFLVHAKRG-APGST

AAS89989.1|Saguinus_oedipus SLLLGRLPTLLRLGRLALWGLGMCCYVFSAQQLQAAQISPDDISLGFLVRAKGV-MPESM

Q53B88.1|Hylobates_lar SLLRGRLPTLLHLGRLALWGLGMCCYVFSAQQLQAAQVSPDDISLGFLVRAKGV-VPGST

NP_071445.1|Homo_sapiens 538 SLLRGRLPTLLHLGRLALWGLGMCCYVFSAQQLQAAQVSPDDISLGFLVRAKGV-VPGST 596

NP_001098710.1|Pan_troglodytes SLLRGRLPTLLHLGRLALWGLGMCCYVFSAQQLQAAQVSPDDISLGFLVRAKGV-VPGST

AAY97879.1|Danio_rerio AWLAEHLDTVLKLGELALEGLQTSCYVFSGYELQRNRITEQDVGIGFLIYCSDI-SVNDC

ACX71753.1|Ctenopharyngodon_idel AWLEEHLDTVLKLGQLALEGLEASCYVFSGNELQRNGLTEQDVSMGFLIYCNDL-SVTDC

AFY26969.1|Carassius_auratus AWLEEHLNTVLKLGQLALEGLEASCYVFSGYELQRIGLTDQDVSMGFLIYCSDL-SVTDC

AEG89706.1|Labeo_rohita AWLEEHLDTVLKLGQLALEGLKASCYVFSGYELQRNGPTEQDVSMGFLIYCNDL-SATDC

ADV31549.1|Oncorhynchus_mykiss GWVQEHHDTALHLGQLAMEGLGASCYIFTGTKLQKCGVTEEDICLGFLIRSKNLSSSTNC

NP_001035913.1|Takifugu_rubripes SWHREYLDPVLRLGQLAFKGIVSTCYIFSDKDLDPCAITKEDLSLGFLIQSKDL-SVFRS

AFV53358.1|Epinephelus_coioides GWLQEHLKTVLHLGQLAFEGLRTSCYMFSDTDLETCGVTEKDICMGFLIQSKEM-SSTHS

AJF23836.1|Larimimichthys_crocea GWFQDHLKTVLHLGQLAFEGMKNTCYIFSGTDLETCGVTENDIYMGFLIQSKDL-SSNHG

. *.** **: *: **:*: .*: . : :***: ..

ERE77543.1|Cricetulus_griseus TPLEFLHITFQCFFAAFYLAVSADTSAASLRHLFSCG-RLGSSLVVRLLPTLCIQGSRVR

NP_001099642.1|Rattus_norvegicus APLEFLHITFQCFFAAFYLAVSADTSAASLKHLFSCG-RPGNSLLLRLLPNLCIQGSRVK

AAN52938.1|Mus_spretus APLEFLHITFQCFFAAFYLAVSADTSVASLKHLFSCG-RLGSSLLGRLLPNLCIQGSRVK

AAN52482.1|Mus_musculus_domestic ASLEFLHITFQCFFAAFYLAVSADTSAASLKHLFSCG-RLGSSLLGRLLPNLCIQGSRVK

AAN52483.1|Mus_musculus_castaneu APLEFLHITFQCFFAAFYLAVSADTSVASLKHLFSCG-RLGSSLLGRLLPNLCIQGSRVK

Q8K3Z0.1|Mus_musculus APLEFLHITFQCFFAAFYLAVSADTSVASLKHLFSCG-RLGSSLLGRLLPNLCIQGSRVK

AAN52480.1|Mus_musculus_musculus APLEFLHITFQCFFAAFYLAVSADTSVASLKHLFSCG-RLGSSLLGRLLPNLCIQGSRVK

EHB15612.1|Heterocephalus_glaber APLEFLHITFQCFFAAFYLVLCADVPTASLRHLFKCH-RPGISQLTRLLPALCVQSPRTK

KFO37911.1|Fukomys_damarensis ATLEFLHITFQCFFAAFYLVLCADVPTASLRHLFKCH-RASSSQLTRLLPALCVQSPRTK

BAH24204.1|Sus_scrofa PPLEFLHVTFQCFFAAFYLALSADVPPSSLRHLFHGH-RPGSSPLAKVLPKLCVRGSGCK

AHL44890.1|Capra_hircus APLEFLHITFQCFFAAFYLALSADTLPSSLRHLFQGH-RPGSSPLARVLPKWFLRGSRCK

AJC01046.1|Bubalus_bubalis APLEFLHITFQCFFAAFYLALSADTPPSSLRHLFQGH-RPGSSPLARVLPKLFLRGSRCR

ELR51388.1|Bos_mutus APLEFLHITFQCFFAAFYLALSADTPPSSLRHLFQDH-RPESSPLARVLPKLFLRGSRCR

AAS09828.1|Bison_bison APLEFLHITFQCFFAAFYLALSADTPPSSLRHLFQDH-RPESSPLARVLPKLFLRGSRCR

AAS09827.1|Bos_indicus APLEFLHITFQCFFAAFYLALSADTPPSSLRHLFQDH-RPESSPLARVLPKLFLRGSRCR

NP_001002889.1|Bos_taurus APLEFLHITFQCFFAAFYLALSADTPPSSLRHLFQDH-RPESSPLARVLPKLFLRGSRCR

NP_001273968.1|Canis_lupus_famil TPLEFLHITFQCFFAALYLVLSTDVSPSLLRQLFICH-GPRSSLLARLLPTTCVPRSERK

ELK15377.1|Pteropus_alecto APLEFLHITFQCFFAAFYLALSAELPASLLRHLFGGR-QPGSSLLARLLSTLCVPDSRCE

AAS89989.1|Saguinus_oedipus APLEFLHITFQCFFAAFYLTLSADVPPALLRHLFNCG-RPGNSLMARFLPMLCIQGSRGK

Q53B88.1|Hylobates_lar APLEFLHITFQCFFAAFYLALSADVPPALLRHLFNCG-RPGNSPVARLLPTLCIQGSEGK

NP_071445.1|Homo_sapiens 597 APLEFLHITFQCFFAAFYLALSADVPPALLRHLFNCG-RPGNSPMARLLPTMCIQASEGK 655

NP_001098710.1|Pan_troglodytes APLEFLHITFQCFFAAFYLALSADVPPALLRHLFNCG-RPGNSPMARLLPTMCIQGSEGK

AAY97879.1|Danio_rerio KRYEFLHITLQCFFAALYVILNRNNDRSAISRLFQPR-NRQVSGLSQSCLGQCM-----D

ACX71753.1|Ctenopharyngodon_idel KHYEFLHITLQCFFAALYVVLNCNNNCSAISRLFQPQ-HRQLSGLSQRCLGHCI-----D

AFY26969.1|Carassius_auratus KRYEFLHITLQCFFAALYVVLNRNRYRSAIYRLFQPK-YKQLAGLSQRCLEPCF-----D

AEG89706.1|Labeo_rohita KSYEFIHVTLQRFFAALYVVLNRNGDRSAISRLFQPQ-YRQLSGRSQRCLGHCI-----D

ADV31549.1|Oncorhynchus_mykiss KHYEFLHITMQCFFAALYIVLNNNSDRSTIPKLFHLQDRQQQPGFHRACLGHCL---TQQ

NP_001035913.1|Takifugu_rubripes KCYEFLHVTMQCFFAAVYVVVSKDLDRSTIPKLFELK-NLKETSLRTACFSACLLYQQVD

AFV53358.1|Epinephelus_coioides KRYEFLHLTMQCFFAALYIVLSNNTERAAIPKLFELQ-NMKETGLSSTCLTACLPSTNQE

AJF23836.1|Larimimichthys_crocea KHYEFLHVTMQCFFAALYIVLSNNMDHSTISKLFEVK-DMWETDLTSSCFRACLPSTYQQ

**:*:*:* ****.*: : : : : .** . .

ERE77543.1|Cricetulus_griseus KGSKAALLQKAEPHNLQITAAFLAGLLSKEHWDLLAACQISKKVLLQRQACARSCLAQSL

NP_001099642.1|Rattus_norvegicus -GGQAALLQKAEPHNLQITAAFLAGLLSQQHRDLLAACQVPERVLLQRQARARSCLAHSL

AAN52938.1|Mus_spretus KGSEAALLQKAEPHNLQITAAFLAGLLSQQHRDLLAACQISERVLLQRQARARSCLAHSL

AAN52482.1|Mus_musculus_domestic KGSEAALLQKAEPHNLQITAAFLAGLLSQQHRDLLAACQISERVLLQRQARARSCLAHSL

AAN52483.1|Mus_musculus_castaneu KGSEAALLQKAEPHNLQITAAFLAGLLSQQHRDLLAACQISERVLLQRQARARSCLAHSL

Q8K3Z0.1|Mus_musculus KGSEAALLQKAEPHNLQITAAFLAGLLSQQHRDLLAACQVSERVLLQRQARARSCLAHSL

AAN52480.1|Mus_musculus_musculus KGSEAALLQKAEPHNLQITAAFLAGLLSQQHRDLLAACQVSERVLLQRQARARSCLAHSL

EHB15612.1|Heterocephalus_glaber EGSAAALLQKAEPHNLQITAAFLAGLLSREHLDLLAECQVPEKALLRRQACARWCLARSL

KFO37911.1|Fukomys_damarensis EGSVTALLQKAEPHNLQITAAFLAGLLSREHLDLLAECQVSEKALLRRQTCARWCLARSL

BAH24204.1|Sus_scrofa KSSVAHLLQEAEPHNLQITAAFLAGLLSQEHRGLLAECQVSEKALLQRQACARWCLARSL

AHL44890.1|Capra_hircus EGSVAALLQGAEPHNLQITGAFLAGLLSQEHRSMLAECQGSETALLRRWDCVRRCLTRSL

AJC01046.1|Bubalus_bubalis EGSVAALLQGAEPHNLQITGAFLAGLLSQEHRSLLAECQASETGLLRRWDCVRQCLTRSL

ELR51388.1|Bos_mutus EGSVAALLQGAELHNLQITGAFLAGLLSQEHRSLLAECQASETALLRRWDCVRRCLTRSL

AAS09828.1|Bison_bison EGSVAALLQGAEQHNLQITGAFLAGLLSQEHRSLLAECQASETALLRRWDCVRRCLTRSL

AAS09827.1|Bos_indicus EGSVAALLQGAEPHNLQITGAFLAGLLSQEHRSLLAECQASETALLRRWDCVQRCLTRSL

NP_001002889.1|Bos_taurus EGSVAALLQGAEPHNLQITGAFLAGLLSQEHRSLLAECQASETALLRRWDCVRRCLTRSL

NP_001273968.1|Canis_lupus_famil EGSLAALLQEAEPHNLQITAAFLAGLLSREHRGLLAECQANEEALLRLQGRAQGCLSRSL

ELK15377.1|Pteropus_alecto EASVAALLQEAEPHNLQITAAFLAGLVSREHRVLLAECQASEKALLRRQACARRCLARSL

AAS89989.1|Saguinus_oedipus DGSLAALLQKTEPHNLQITAAFLAGLLSWEHWGLLAECQASEKALLQRQACARWCLARSL

Q53B88.1|Hylobates_lar DSSVAALLQKAEPHNLQITAAFLAGLLSREHWGLLAECQTSEKALLRRQACARWCLARSL

NP_071445.1|Homo_sapiens 656 DSSVAALLQKAEPHNLQITAAFLAGLLSREHWGLLAECQTSEKALLRRQACARWCLARSL 715

NP_001098710.1|Pan_troglodytes DSSVAALLQKAEPHNLQITAAFLAGLLSREHWGLLAECQTSEKALLRRQACARWCLARSL

AAY97879.1|Danio_rerio HSVE--ESHEAETANLQITAQFVSGLLSQRHNNLFLEC-CPAVVRERNVKQVVKSLSKRM

ACX71753.1|Ctenopharyngodon_idel PSVEEAESHVAETPNLQITAQFVSGLLSQRHHSLLLDC-CPTAVRERKFKQVAKSLSKGM

AFY26969.1|Carassius_auratus SSAEGMESRVTETPNLQITAQFVSGLLSQRHHNLLLEC-CPTAMRERKAKQVVKSLSKCM

AEG89706.1|Labeo_rohita SSVEEVESHVTETPNLQITAQFVSGLLSRRHHYLLLEC-CPAAVWERKVKQVVKLLSKGM

ADV31549.1|Oncorhynchus_mykiss EGALKEANKAAETPNLQITATFVSGLLAQRHRSLLLLS-CPGPTLDRKSKQVVTCLSKGI

NP_001035913.1|Takifugu_rubripes ECSLDKDATAVETPNLQMTATFASGLLSQRHRSLWLHC-CPATAIDRKTRQVSKCLSKGM

AFV53358.1|Epinephelus_coioides QASDG-EATAAETPNLQITATFVSGLLSQHHRSLWRHC-CSSAVMEKRVRQVARCLSKGM

AJF23836.1|Larimimichthys_crocea DCSLQGDATAAETSNLQITATFVSGLLSKRHQSLWLQC-CPSAVMEKKIRQVGKCLSKGM

.* ***:*. * :**::. * : . . . *:. :

ERE77543.1|Cricetulus_griseus REHFHSIPPAVPGEAKSMHAMPGFIWLIRSLYEMQEEQLAQEAVRRLDIGHLKLTFCRVG

NP_001099642.1|Rattus_norvegicus REHFHSIPPAVPGETKSMHAMPGFIWLIRSLYEMQEVQLAREAVRRLDIGHLKLTFCRVG

AAN52938.1|Mus_spretus REHFHSIPPAVPGETKSMHAMPGFIWLIRSLYEMQEEQLAQEAVRRLDIGHLKLTFCRVG

AAN52482.1|Mus_musculus_domestic REHFHSIPPAVPGETKSMHAMPGFIWLIRSLYEMQEEQLAQEAVRRLDIGHLKLTFCRVG

AAN52483.1|Mus_musculus_castaneu REHFHSIPPAVPGETKSMHAMPGFIWLIRSLYEMQEEQLAQEAVRRLDIGHLKLTFCRVG

Q8K3Z0.1|Mus_musculus REHFHSIPPAVPGETKSMHAMPGFIWLIRSLYEMQEEQLAQEAVRRLDIGHLKLTFCRVG

AAN52480.1|Mus_musculus_musculus REHFHSIPPAVPGETKSMHAMPGFIWLIRSLYEMQEEQLAQEAVRRLDIGHLKLTFCRVG

EHB15612.1|Heterocephalus_glaber RKHFHCIPPAVPGEAKSMHAMPGFIWLIRSLYEMQEERLAREAVRGLDVEHLKLTFCSVG

KFO37911.1|Fukomys_damarensis RKHFHCIPPAVPGEAKSMHAMPGFVWLIRSLYEMQEERLAREAVRGLDVEHLKLTFCGVG

BAH24204.1|Sus_scrofa HKHFRSIPPAVPGEVKSMHAMPGFIWLIRSLYEMQEERLARDAVSRLNVGHLKLTFCSVG

AHL44890.1|Capra_hircus CKHFRSIPPAVPGEAKSMHALPGFLWLIRSLYEMQEERLAREAARRLNVGHLKLTFCSVG

AJC01046.1|Bubalus_bubalis REHFRCIPPALPGEAKSMHALPGFLWLIRSLYEMQEERLAQEAVRRLNVGHLKLTFCSVG

ELR51388.1|Bos_mutus REHFRSIPPALPGEAKSMHALPGFLWLIRSLYEMQEERLAWEAVRRLNVGHLKLTFCGVG

AAS09828.1|Bison_bison REHFRSIPPALPGEAKSMHALPGFLWLIRSLYEMQEERLAREAVRRLNVGHLKLTFCGVG

AAS09827.1|Bos_indicus HEHFRSIPPALPGEAKSMHALPGFLWLIRSLYEMQEERLAREAVRRLNVGHLKLTFCGVG

NP_001002889.1|Bos_taurus REHFRSIPPALPGEAKSMHALPGFLWLIRSLYEMQEERLAREAVCRLNVGHLKLTFCGVG

NP_001273968.1|Canis_lupus_famil HQHFRSIPPAVPGEAKSMHAMPGFLWLIRSLYEMQEERLAREAVRGLTVGHLKLTFCGVG

ELK15377.1|Pteropus_alecto HEHFHTIPPAVPGEAKSMHAMPGFVWLIRSLYEMREERLAREAVRGLNVGHLKLTFCGVG

AAS89989.1|Saguinus_oedipus RKHFHSIPPAAPGEAKSMHAMPRFIWLLRSLYEMQDERLARKAVRGLNVGHLKLTFCSMG

Q53B88.1|Hylobates_lar RKHFHSIPPAAPGEAKSMHAMPGFIWLIRSLYEMQEERLARKAARGLNVGHLKLTFCSVG

NP_071445.1|Homo_sapiens 716 RKHFHSIPPAAPGEAKSVHAMPGFIWLIRSLYEMQEERLARKAARGLNVGHLKLTFCSVG 775

NP_001098710.1|Pan_troglodytes RKHFHSIPPAAPGEAKSVHAMPGFIWLIRSLYEMQEERLARKAARGLNVGHLKLTFCSVG

AAY97879.1|Danio_rerio QRHFKSIPRPVEGEKKSMHAMPSFVWLIKCIYELQDNSIAQDAMAKLDVEHLKLTYCNIG

ACX71753.1|Ctenopharyngodon_idel QRHFKSIPRPVKGEKKSMHAMPSFVWLIKCIYEMQDKSIAQDAVAKLDVEHLKLTYCSIG

AFY26969.1|Carassius_auratus QRHFKSIPRPLEGEKKSVHAMPSFVWLIKCIYEMQDSSIAQDTMAKLDVEHLKLTYCNIG

AEG89706.1|Labeo_rohita QRHFKSIPRPVEGEKKSMHAMPSFVWLIKCIYEMQDNSIAQDTMAKLDVEHLKLTYCNIG

ADV31549.1|Oncorhynchus_mykiss QKHFKSIPRPVEGEKKSMHAMPGFVWLIKCIHEVQDSRIARDTMAKLEVEHLKLAYCNIG

NP_001035913.1|Takifugu_rubripes QKHFKSIPQPVAGEKKSMHAMPGFVWLIKCIYEMQECSIAKDAMSKLEVDHLKLTYCNIG

AFV53358.1|Epinephelus_coioides QKHFKSIPQAVEGEKKSMHAMPGFVWLIKCIYEMQESRIAKDAMSKLEVDHLKLTYCNIG

AJF23836.1|Larimimichthys_crocea QKHFKSIPQPVEGEKKSMHAMPGFVWLIKCIYEMQESRIAKDAMSKLEVDHLKLTYCNIG

**. ** . ** **:**:* *:**:..::*:.: :*..: * : ****::* :*

ERE77543.1|Cricetulus_griseus PAECAALAFVLRHLQRPVALQLDHNSVGDVGVEQLLPCLGVCTALYLRDNNISDRGVCTL

NP_001099642.1|Rattus_norvegicus PAECAALAFVLQHLQRPVALQLDHNSVGDVGVEQLLPCLGVCTALYLRDNNISDRGARTL

AAN52938.1|Mus_spretus PAECAALAFVLKHLQRPVALQLDYNSVGDVGVEQLRPCLGVCTALYLRDNNISDRGACTL

AAN52482.1|Mus_musculus_domestic PAECAALAFVLQHLQRPVALQLDYNSVGDVGVEQLRPCLGVCTALYLRDNNISDRGARTL

AAN52483.1|Mus_musculus_castaneu PAECAALAFVLQHLQRPVALQLDYNSVGDVGVEQLRPCLGVCTALYLRDNNISDRGARTL

Q8K3Z0.1|Mus_musculus PAECAALAFVLQHLQRPVALQLDYNSVGDVGVEQLRPCLGVCTALYLRDNNISDRGARTL

AAN52480.1|Mus_musculus_musculus PAECAALAFVLQHLQRPVALQLDYNSVGDVGVEQLRPCLGVCTALYLRDNNISDRGARTL

EHB15612.1|Heterocephalus_glaber PTECAALAFVLRHLQQPLALQLDYNSVGDIGVEQLLPCLGVCKALYLRDNNISDRGICKL

KFO37911.1|Fukomys_damarensis PAECAALAFVLQHVQRPLALQLDYNSVGDIGVEQLRPCLGVCKALYLRDNNISDRGICKL

BAH24204.1|Sus_scrofa PAECAALAFVLRHLRWPVALQLDHNSVGDIGVEQLLPCLNVCKALYLRDNNISDRGFCKL

AHL44890.1|Capra_hircus PAECAALAFVLRHLRXPVALQLDHNSVGDIGVEQLLPCLGVCKALYLRDNNISDRGICKL

AJC01046.1|Bubalus_bubalis PAECAALAFVLRHLQRPVALQLDHNSMGDIGVEQLLPCLGXCKALSLRDNNISDRGICKL

ELR51388.1|Bos_mutus PAECAALAFVLRHLRRPVALQLDHNSVGDIGVEQLLPCLGVCKALYLRDNNISDRGICKL

AAS09828.1|Bison_bison PAECAALAFVLRHLRRPVALQLDHNSVGDIGVEQLLPCLGVCKALYLRDNNISDRGICKL

AAS09827.1|Bos_indicus PAECAALAFVLRHLRRPVALQLDHNSVGDIGVEQLLPCLGVCKALYLRDNNISDRGICKL

NP_001002889.1|Bos_taurus PAECAALAFVLRHLRRPVALQLDHNSVGDIGVEQLLPCLGVCKALYLRDNNISDRGICKL

NP_001273968.1|Canis_lupus_famil PPECAALAFVLRHLRRPVALQLDHNSVGDIGVEQLLPCLSVCKALYLRDNNISDRGICKL

ELK15377.1|Pteropus_alecto PAECAALAFVLRHLRQPVALQLDHNSVGDVGVEQLLPCLGVCKALYLRDNNISDRGICKL

AAS89989.1|Saguinus_oedipus PTECAALAFVLRHLRRPVALQLDYNSVGDIGVEQLLPCLGVCKALYLRNSNILDRGICKL

Q53B88.1|Hylobates_lar PAECAALAFVLQHLRRPVALQLDYNSVGDIGVEQLLPCLGVCKALYLRDNNISDRGICKL

NP_071445.1|Homo_sapiens 776 PTECAALAFVLQHLRRPVALQLDYNSVGDIGVEQLLPCLGVCKALYLRDNNISDRGICKL 835

NP_001098710.1|Pan_troglodytes PAECAALAFVLQHLRRPVALQLDYNSVGDIGVEQLLPCLGVCKALYLRDNNISDRGICKL

AAY97879.1|Danio_rerio PVECTALAYVLKNLRKPVGLQLDNNSVGDVGVEQLLPCLPMCHSLYLRNNNISDEGIRKL

ACX71753.1|Ctenopharyngodon_idel PVECTALAYVLQHLRNPVGLQLDNNSVGDVGVEQLLPCLHICHSLYLRNNNISDEGIRKL

AFY26969.1|Carassius_auratus PVECTALAYVLKYLRNPVGLQLDNNSVGDVGAEQLLPCLHICHSLYLRNNNISDEGIRKL

AEG89706.1|Labeo_rohita PVECTALAYVLKYLRNPVGLQLDNNSVGDVGVEQLLPCLHICHSLYLRNNNISDEGIRKL

ADV31549.1|Oncorhynchus_mykiss PGECTALAYVLQHLRNPVGLQLDYNYVGDVGVEQLLPCLHVCHSVYLRHNNISDEGVRKL

NP_001035913.1|Takifugu_rubripes PVECTALAFVLQHLNNPVGLQLDNNTVGDVGVEQLLPCMHICHSLYLRNNNITDEGIHKL

AFV53358.1|Epinephelus_coioides PVECTALAYVLQHLKNPVGLQLDNNSVGDVGVEQLLPCMHICNSLHLRNNNITDEGIRKL

AJF23836.1|Larimimichthys_crocea PVECTALAYVLQHLRNPVGLQLDNNSVGDVGVEQLLPCMHICNSLYLRNNNITDEGIRKL

* **:***:**. :. *:.**** * :**:*.*** **: * :: ** .** * * .*

ERE77543.1|Cricetulus_griseus IEYALHCEQLQKLALFNNKLTDGCAYSMAKLLAHRQNFLSLRVGNNHITAAGAQVLAQGL

NP_001099642.1|Rattus_norvegicus VECALRCEQLQKLALFNNKLTDGCASSVAKLLAHKQNFLSLRVGNNHITAAGAEVLAQGL

AAN52938.1|Mus_spretus VECALRCEQLQKLALFNNKLTDACACSMAKLLAHKQNFLSLRVGNNHITAAGAEVLAQGL

AAN52482.1|Mus_musculus_domestic VECALRCEQLQKLALFNNKLTDACACSMAKLLAHKQNFLSLRVGNNHITAAGAEVLAQGL

AAN52483.1|Mus_musculus_castaneu VECALRCEQLQKLALFNNKLTDACACSMAKLLAHKQNFLSLRVGNNHITAAGAEVLAQGL

Q8K3Z0.1|Mus_musculus VECALRCEQLQKLALFNNKLTDACACSMAKLLAHKQNFLSLRVGNNHITAAGAEVLAQGL

AAN52480.1|Mus_musculus_musculus VECALRCEQLQKLALFNNKLTDACACSMAKLLAHKQNFLSLRVGNNHITAAGAEVLAQGL

EHB15612.1|Heterocephalus_glaber IEYALHYGQLQKLALFNNRLTDGCAHSMAKLLECKQNFLALRLGNNHITAAGAQVLAQGL

KFO37911.1|Fukomys_damarensis IEHALHYGQLQKLALFNNKLTDGCTHSMAKLLECKQNFLALRLGNNHITAAGAQVLAQGL

BAH24204.1|Sus_scrofa VEHALRCEQLQKLALFNNKLTDGCAHSMARLLACKRNFLALRLGNNHFTAVGAQVLAQGL

AHL44890.1|Capra_hircus VEHALRCEQLQKLALFNNKLTDACTHSMARLLACKQNFLALRLGNNHITAAGAEVLAQGL

AJC01046.1|Bubalus_bubalis VEHALHCEQLQKLALFNNKLTDGCAHSMARLLACKQNFLALRLGNNHITAAGAEVLAQGL

ELR51388.1|Bos_mutus VEHALRCEQLQKLALFNNKLTDGCAHSMARLLACKQNFLALRLGNNHITAAGAEVLAQGL

AAS09828.1|Bison_bison VEHALRCEQLQKLALFNNKLTDGCAHSMARLLACKQNFLALRLGNNHITAAGAEVLAQGL

AAS09827.1|Bos_indicus VEHALRCEQLQKLALFNNKLTDGCAHSMARLLACKQNFLALRLGNNHITAAGAEVLAQGL

NP_001002889.1|Bos_taurus VEHALRCEQLQKLALFNNKLTDGCAHSMARLLACKQNFLALRLGNNHITAAGAEVLAQGL

NP_001273968.1|Canis_lupus_famil IEHALHCEQLQKLALFNNKLTDGCAHSMARLLACKQNFLALRLGNNRITAAGAQALAEGL

ELK15377.1|Pteropus_alecto IEHALHCEKLQKLAL----------------------------GNNHITAAGAQVLAQGL

AAS89989.1|Saguinus_oedipus IEHALHCEPLWKLVLFNNKLTDGCAHSMAKLLACKQNFLALRLGNNYITAAGAQVLAEGL

Q53B88.1|Hylobates_lar IECALHCEQLQKLVLFNNKLTDGCAHSMAKLLACRQNFLALRLGNNHITPAGAQVLAEGL

NP_071445.1|Homo_sapiens 836 IECALHCEQLQKLALFNNKLTDGCAHSMAKLLACRQNFLALRLGNNYITAAGAQVLAEGL 895

NP_001098710.1|Pan_troglodytes IECALHCEQLQKLALFNNKLTDGCAHSMAKLLACRQNFLALRLGNNYITAAGAQVLAQGL

AAY97879.1|Danio_rerio LEKGIECENFQKIALFNNKLTDTCTQYFSCLLKSKQNFLALRLGNNNITSVGAEQLAEGL

ACX71753.1|Ctenopharyngodon_idel LEKGVKCESFQKIALFNNKLTDACTQHFACLLKTKQNFLALRLGNNNITSQGAEQLAEGL

AFY26969.1|Carassius_auratus LEKGMKCERFQKIALFNNNLTDACTQHFAWLLKSKQNFLSLRLGNNNITSQGAEQLAEGL

AEG89706.1|Labeo_rohita LEKGMTCESFQKIALFNNNLTDACTQHFALLLKSKQNFLALRLGNNNITSQGAEQLAEGL

ADV31549.1|Oncorhynchus_mykiss IEKGIQCERFQKIALFNNKLTDACTECFAHLLKMKQNFLSLRLGNNNITAAGAGQLAEGL

NP_001035913.1|Takifugu_rubripes IAKSIQCDNFHKIALFNNRLTDACTQDFSLLLKTKQDFISLRLGNNNITAEGAKQLAEGL

AFV53358.1|Epinephelus_coioides LAKGIQCCDFQKIALFNNKLTDACTQHFSHLLKTKQDFLALRLGNNNITEEGAKQLAEGL

AJF23836.1|Larimimichthys_crocea IAKGIQCENFRKIALFNNKLTDACTQHISQLLRTKDDFLSLRLGNNNITAEGAKQLAEGL

: .: :.*:.* *** :* ** **:**

ERE77543.1|Cricetulus_griseus KSNTSLQFLGFWGNSVGDKGTQALAEALAGHQSIKWLSLVGNNIGSEGAQALAMMLEKNK

NP_001099642.1|Rattus_norvegicus KSNTSLQFLGFWGNSVGDKGSQALAEVVADHQRLKWLSLVGNNIGSVGAHALALMLEKNK

AAN52938.1|Mus_spretus KSNTSLKFLGFWGNSVGDKGTQALAEVVADHQNLKWLSLVGNNIGSMGAQALALMLEKNK

AAN52482.1|Mus_musculus_domestic KSNTSLKFLGFWGNSVGDKGTQALAEVVADHQNLKWLSLVGNNIGSMGAQALALMLEKNK

AAN52483.1|Mus_musculus_castaneu KSNTSLKFLGFWGNSVGDKGTQALAEVVADHQNLKWLSLVGNNIGSMGAQALALMLEKNK

Q8K3Z0.1|Mus_musculus KSNTSLKFLGFWGNSVGDKGTQALAEVVADHQNLKWLSLVGNNIGSMGAEALALMLEKNK

AAN52480.1|Mus_musculus_musculus KSNTSLKFLGFWGNSVGDKGTQALAEVVADHQNLKWLSLVGNNIGSMGAEALALMLEKNK

EHB15612.1|Heterocephalus_glaber RSNASLQFLGFWGNTVGDKGAQALAEALVDHHSLRWLSLVGNNIGSVGAQALAQMLEKNM

KFO37911.1|Fukomys_damarensis RSNASLQFLGFWGNTVGDKGAQALAEALGDHQSLRWLSLVGNNIGSMGARALAQMLEKNM

BAH24204.1|Sus_scrofa RANTSLQFLGFWGNQVGDKGAQALAEALRDNQSLKWLSLVGNNIGSVGAQALALMLEKNM

AHL44890.1|Capra_hircus RTNNSLQFLGFWGNQVGDEGAQALAAALGDHQSLRWLSLVGNNIGSVGAQALALMLEKNM

AJC01046.1|Bubalus_bubalis RTNNSLQFLGFWGNQVGDEGAQALAAALGDHQSLRWLSLVGNNIGSVGAQALALMLEKNM

ELR51388.1|Bos_mutus RTNNSLQFLGFWGNQVGDEGAQALAAALGDHQSLRWLSLVGNNIGSVGAQALALMLEKNM

AAS09828.1|Bison_bison RTNNSLQFLGFWGNQVGDEGAQALAAALGDHQSLRWLSLVGNNIGSVGAQALALMLEKNM

AAS09827.1|Bos_indicus RTNNSLQFLGFWGNQVGDEGAQALAAALGDHQSLRWLSLVGNNIGSVGAQALALMLEKNM

NP_001002889.1|Bos_taurus RTNNSLQFLGFWGNQVGDEGAQALAAALGDHQSLRWLSLVGNNIGSVGAQALALMLEKNM

NP_001273968.1|Canis_lupus_famil RANTSLQFLGFWGNKVGDEGAQALAEALGDHQSLRWLSLVGNDIGSVGARALALMLEKNV

ELK15377.1|Pteropus_alecto RANASLQFLGFWGNKVGDEGAQALAEAVGDHQSLKWLSLVGNDIGSTGARALASMLEKNV

AAS89989.1|Saguinus_oedipus RGNTSLQFLGFWGNRMGDEGAQALAEALSDHQSLRWLSLVGNNIGSVGAQALALMLAKNV

Q53B88.1|Hylobates_lar RGNTSLQFLGFWGNRVGDEGAQALAEALGDHQSLRWLSLVGNNIGSVGAQALALMLAKNV

NP_071445.1|Homo_sapiens 896 RGNTSLQFLGFWGNRVGDEGAQALAEALGDHQSLRWLSLVGNNIGSVGAQALALMLAKNV 955

NP_001098710.1|Pan_troglodytes RGNTSLQFLGFWGNRVGDEGAQALAEALGDHQSLRWLSLVGNNIGSVGAQALALMLAKNV

AAY97879.1|Danio_rerio SYNQSLQFLGLWGNKVGDRGAEVLADALTNSKTLIWLSLVDNGVGSAGACALAKFIRQNK

ACX71753.1|Ctenopharyngodon_idel SYNQSLQFLGLWGNKIGDRGAEALANALKNSTTLIWLSLVDNGVGSAGACALAKLISQSK

AFY26969.1|Carassius_auratus SYNQSLQFLGLWGNKIGDRGAEALADALKNSTTLIWLSLVDNGVGSAGACALAKIISQSK

AEG89706.1|Labeo_rohita SYNQSLQFLGLWGNKIGDRGAEALASALKNSTSLIWLSLVDNGVGSAGACALAELISQSK

ADV31549.1|Oncorhynchus_mykiss RFNRSLQFLGLWGNKIGDKGAEAIANALKDSQTLVWLSLVDNGVGNAGACALAPLI-KNS

NP_001035913.1|Takifugu_rubripes RVNKSLKFLGLWGNSIGDAGAEALASALEGNTTLVWLSLVGNGIGSAGACALSKVVKNNV

AFV53358.1|Epinephelus_coioides KLNHSLQYLGLWGNRIGDAGAEALASALENSKTLIWLSLVGNGVGSAGACALANIIKNST

AJF23836.1|Larimimichthys_crocea KFNHSLQYLGLWGNKIGDTGAEALAKALESSKTLVWLSLVGNGVGNAGASALANIIKNSP

* **::**:*** :** *::.:* .: . : *****.*.:*. ** **: .: :.

ERE77543.1|Cricetulus_griseus SLEELCLEENHVCDEGVYFLTEGLKRNSSLKILKLSNNGITYGGAEALLQALDRNSTILE

NP_001099642.1|Rattus_norvegicus SLEE--------------------------------------------------------

AAN52938.1|Mus_spretus SLEELCLEENHICDEGVYSLAEGLKRNSTLKFLKLSNNGITYRGAEALLQALSRNSAILE

AAN52482.1|Mus_musculus_domestic SLEELCLEENHICDEGVYSLAEGLKRNSTLKFLKLSNNGITYRGAEALLQALSRNSAILE

AAN52483.1|Mus_musculus_castaneu SLEELCLEENHICDEGVYSLAEGLKRNSTLKFLKLSNNGITYRGAEALLKALSRNSAILE

Q8K3Z0.1|Mus_musculus SLEELCLEENHICDEGVYSLAEGLKRNSTLKFLKLSNNGITYRGAEALLQALSRNSAILE

AAN52480.1|Mus_musculus_musculus SLEELCLEENHICDEGVYSLAEGLKRNSTLKFLKLSNNGITYRGAEALLQALSRNSAILE

EHB15612.1|Heterocephalus_glaber ALEE----------------------------LWLSNNSVTYRGAEALLQALESNNTILE

KFO37911.1|Fukomys_damarensis ALEE----------------------------LWLSNNNVTYRGAEALLQTLESNNTILE

BAH24204.1|Sus_scrofa ALEELCLEENHVQDEGVCSLARGLERNSSLKVLKLSNNHVTSRGAEALLQALEQNDTILE

AHL44890.1|Capra_hircus ALEELCLEENHVQDEGVCFLAKGLARNSSLKVLKLSNNHITSLGAEALLWALEKNDTILE

AJC01046.1|Bubalus_bubalis ALEELCLEENHVQDEGVCFLAKGLARNSSLKVLKLSNNHITSLGAEALLRALEKNDTILE

ELR51388.1|Bos_mutus ALEELCLEENHVQDEGVCFLAKGLARNSSLKVLKLSNNHISSLGAEALLWALEKNDTILE

AAS09828.1|Bison_bison ALEELCLEENHVQDEGVCFLAKGLARNSSLKVLKLSNNHISSLGAEALLRALEKNDTILE

AAS09827.1|Bos_indicus ALEELCLEENHVQDEGVCFLAKGLARNSSLKVLKLSNNHISSLGAEALLRALEKNDTILE

NP_001002889.1|Bos_taurus ALEELCLEENHVQDEGVCFLAKGLARNSSLKVLKLSNNHISSLGAEALLRALEKNDTILE

NP_001273968.1|Canis_lupus_famil ALEELCLEENHLQDEGVCSLAKGLERNSSLKVLKLSNNCITYLGAEGLLQALEKNDTILE

ELK15377.1|Pteropus_alecto ALEELCLEENHLQDEGVCSLAEGLKRNSSLKVLKLSNNRITCRGAEALLKALERNDTILE

AAS89989.1|Saguinus_oedipus MLEELCLEENHLQDEGVCSLAEGLKKNSSLKILKLSNNCITYLGAEALLQALERNDTILE

Q53B88.1|Hylobates_lar MLEELCLEENHIQDEGVCSLAEGLKKNSSLKILKLSNNCITYLGAKALLQALERNDTILE

NP_071445.1|Homo_sapiens 956 MLEELCLEENHLQDEGVCSLAEGLKKNSSLKILKLSNNCITYLGAEALLQALERNDTILE 1015

NP_001098710.1|Pan_troglodytes MLEELCLEENHLQDEGVCSLAEGLKKNSSLKILKLSNNCITYLGAEALLQALERNDTILE

AAY97879.1|Danio_rerio SLEE----------------------------LWLNKNSICKEGVDCLIEALKMNTSVKK

ACX71753.1|Ctenopharyngodon_idel TLDE----------------------------LWLNKNCISRDGVECLIEALKMNSSVRE

AFY26969.1|Carassius_auratus SLEE----------------------------LWLNKNCISREGVECLIEALKVNTSVKE

AEG89706.1|Labeo_rohita TMQE----------------------------LWLNKNCITREGVECLIEALKMNTSIKE

ADV31549.1|Oncorhynchus_mykiss TLEE----------------------------LWLTKNCITRTGVECLILALESNTSVKA

NP_001035913.1|Takifugu_rubripes SLEE----------------------------LWLTENCITRMGVECLIEALQHNANVKS

AFV53358.1|Epinephelus_coioides SLEE----------------------------LWLTQNQITRTGVECLIQALEHNTHVKS

AJF23836.1|Larimimichthys_crocea TLEE----------------------------LWLTENCITRTGVERLIEALKHNIHVKS

::*

ERE77543.1|Cricetulus_griseus VWLRGNTFSLEEIETLSSSDSRLLL--------------------------------

NP_001099642.1|Rattus_norvegicus --L------------------------------------------------------

AAN52938.1|Mus_spretus VWLRGNTFSLEEIQTLSSRDARLLL--------------------------------

AAN52482.1|Mus_musculus_domestic VWLRGNTFSLEEIQTLSSRDARLLL--------------------------------

AAN52483.1|Mus_musculus_castaneu VWLRGNTFSLEEIQTLSSRDARLLL--------------------------------

Q8K3Z0.1|Mus_musculus VWLRGNTFSLEEIQTLSSRDARLLL--------------------------------

AAN52480.1|Mus_musculus_musculus VWLRGNTFSLEEIQTLSSRDARLLL--------------------------------

EHB15612.1|Heterocephalus_glaber VWLRGNTFSPEEIERLSHRDTRLLL--------------------------------

KFO37911.1|Fukomys_damarensis VW-------------------------------------------------------

BAH24204.1|Sus_scrofa VWLRGNNFSPEETEQLSQRDTRLLL--------------------------------

AHL44890.1|Capra_hircus VWLRGNTFSPEEIEKLSHQDTRLLL--------------------------------

AJC01046.1|Bubalus_bubalis VWLRGNTFCPEEIEKLSHQDTRLLL--------------------------------

ELR51388.1|Bos_mutus VWLRGNTFSPEEIEKLSHQDTRLLL--------------------------------

AAS09828.1|Bison_bison VWLRGNTFSPEEIEKLSHQDTRLLL--------------------------------

AAS09827.1|Bos_indicus VWLRGNTFSPEEIEKLSHQDTRLLL--------------------------------

NP_001002889.1|Bos_taurus VWLRGNTFSPEEIEKLSHQDTRLLL--------------------------------

NP_001273968.1|Canis_lupus_famil VWLRGNTFSLEEMERLSQKDTRLLL--------------------------------

ELK15377.1|Pteropus_alecto VWLLRVALSVGSLCWWREVLSRPLFLIEDGRRVETGLASLSCEHSSPFAGATFLKPS

AAS89989.1|Saguinus_oedipus VWLRGNTFSLEEVDKLSCRDTRLLL--------------------------------

Q53B88.1|Hylobates_lar VWLRGNIFSLEEVDKLGCRDIRLLL--------------------------------

NP_071445.1|Homo_sapiens 1016 VWLRGNTFSLEEVDKLGCRDTRLLL-------------------------------- 1040

NP_001098710.1|Pan_troglodytes VWLRGNTFSLEEVDKLGCRDTRLLL--------------------------------

AAY97879.1|Danio_rerio VWLRGNNLSPQEEEQLSQQECRLTF--------------------------------

ACX71753.1|Ctenopharyngodon_idel VWLRGNNLSPEEEVELSKQESRLIF--------------------------------

AFY26969.1|Carassius_auratus IWLRGNNLSPGEEEELSKQESRLTF--------------------------------

AEG89706.1|Labeo_rohita VWLKGNNLRPEEEEELSKQESRLTF--------------------------------

ADV31549.1|Oncorhynchus_mykiss VWLRGNELSPEEVEEMTQREPRLTF--------------------------------

NP_001035913.1|Takifugu_rubripes IWLRNNDLCLEEVEEMAQRESRLLF--------------------------------

AFV53358.1|Epinephelus_coioides VWLRNNDLSLEEVEEMAQRESRLIF--------------------------------

AJF23836.1|Larimimichthys_crocea IWLRNNDLSLEEVEEMTQRESRLVF--------------------------------

**Supplementary File 2: MUSCLE-based alignment of thirty NOD2 protein sequences from different species.** Sequences were submitted to MUSCLE for multiple alignment using standard parameters. Gaps are represented by a hyphen. Accession numbers are given to the left of the alignment. Residue numbering is provided for the human sequence at the start and finish of each line in the alignment. The positions of residues showing polymorphic variation and that were examined as part of this work are highlighted in green on the human sequence. D379, the Walker B residue mutated in the control construct, is highlighted in blue.
